# Landscape of immune-related signatures induced by targeting of different epigenetic regulators in melanoma: implications for immunotherapy

**DOI:** 10.1186/s13046-022-02529-5

**Published:** 2022-11-17

**Authors:** Andrea Anichini, Alessandra Molla, Gabriella Nicolini, Valentina Eleonora Perotti, Francesco Sgambelluri, Alessia Covre, Carolina Fazio, Maria Fortunata Lofiego, Anna Maria Di Giacomo, Sandra Coral, Antonella Manca, Maria Cristina Sini, Marina Pisano, Teresa Noviello, Francesca Caruso, Silvia Brich, Giancarlo Pruneri, Andrea Maurichi, Mario Santinami, Michele Ceccarelli, Giuseppe Palmieri, Michele Maio, Roberta Mortarini, On behalf of the EPigenetic Immune-oncology Consortium AIRC (EPICA) investigators

**Affiliations:** 1 Human Tumors Immunobiology Unit, Department of Research, Fondazione IRCCS Istituto Nazionale dei Tumori, Via Venezian 1, 20133 Milan, Italy; 2 Center for Immuno-Oncology, University Hospital of Siena, Siena, Italy; 3 University of Siena, Siena, Italy; 4 Unit of Cancer Genetics, National Research Council (CNR), Sassari, Italy; 5 Department of Electrical Engineering and Information Technology (DIETI), University of Naples “Federico II”, Naples, Italy; 6 BIOGEM Institute of Molecular Biology and Genetics, Ariano Irpino, Italy; 7 Department of Pathology and Laboratory Medicine, Fondazione IRCCS Istituto Nazionale dei Tumori, Milan, Italy; 8 University of Milan, School of Medicine, Milan, Italy; 9 Melanoma and Sarcoma Unit, Department of Surgery, Fondazione IRCCS Istituto Nazionale dei Tumori, Milan, Italy; 10 University of Sassari, Sassari, Italy

**Keywords:** Melanoma, Epigenetic drugs, Immune-related signatures, Guadecitabine, Innate immunity

## Abstract

**Background:**

Improvement of efficacy of immune checkpoint blockade (ICB) remains a major clinical goal. Association of ICB with immunomodulatory epigenetic drugs is an option. However, epigenetic inhibitors show a heterogeneous landscape of activities. Analysis of transcriptional programs induced in neoplastic cells by distinct classes of epigenetic drugs may foster identification of the most promising agents.

**Methods:**

Melanoma cell lines, characterized for mutational and differentiation profile, were treated with inhibitors of DNA methyltransferases (guadecitabine), histone deacetylases (givinostat), BET proteins (JQ1 and OTX-015), and *enhancer of zeste homolog 2 (GSK126*). Modulatory effects of epigenetic drugs were evaluated at the gene and protein levels. Master molecules explaining changes in gene expression were identified by Upstream Regulator (UR) analysis. Gene set enrichment and IPA were used respectively to test modulation of guadecitabine-specific gene and UR signatures in baseline and on-treatment tumor biopsies from melanoma patients in the Phase Ib NIBIT-M4 Guadecitabine + Ipilimumab Trial. Prognostic significance of drug-specific immune-related genes was tested with Timer 2.0 in TCGA tumor datasets.

**Results:**

Epigenetic drugs induced different profiles of gene expression in melanoma cell lines. Immune-related genes were frequently upregulated by guadecitabine, irrespective of the mutational and differentiation profiles of the melanoma cell lines, to a lesser extent by givinostat, but mostly downregulated by JQ1 and OTX-015. GSK126 was the least active drug. Quantitative western blot analysis confirmed drug-specific modulatory profiles. Most of the guadecitabine-specific signature genes were upregulated in on-treatment NIBIT-M4 tumor biopsies, but not in on-treatment lesions of patients treated only with ipilimumab. A guadecitabine-specific UR signature, containing activated molecules of the TLR, NF-kB, and IFN innate immunity pathways, was induced in drug-treated melanoma, mesothelioma and hepatocarcinoma cell lines and in a human melanoma xenograft model. Activation of guadecitabine-specific UR signature molecules in on-treatment tumor biopsies discriminated responding from non-responding NIBIT-M4 patients. Sixty-five % of the immune-related genes upregulated by guadecitabine were prognostically significant and conferred a reduced risk in the TCGA cutaneous melanoma dataset.

**Conclusions:**

The DNMT inhibitor guadecitabine emerged as the most promising immunomodulatory agent among those tested, supporting the rationale for usage of this class of epigenetic drugs in combinatorial immunotherapy approaches.

**Supplementary Information:**

The online version contains supplementary material available at 10.1186/s13046-022-02529-5.

## Introduction

Immune checkpoint blockade (ICB) has transformed management of a wide range of solid tumors [1]. Antibodies targeting CTLA-4 or the PD-1/PD-L1 axis have been approved for the treatment of 19 cancer types and two tissue-agnostic conditions [2]. Meta-analysis of 23,760 patients and 37 phase II/III trials indicated that ICB provides a survival advantage compared to control treatments, irrespective of age, sex and ECOG performance status [3]. However, only a fraction of patients obtain durable complete responses and both intrinsic and acquired resistance to ICB is frequent [4]. Therefore, there is an urgent need to design improved immunotherapeutic approaches. One option is the association of ICB with immunomodulatory drugs [5]. Inhibitors targeting epigenetic regulators have immune-related effects on tumor cells, as well as on immune cells, that could potentially synergize with ICB [5–8]. Preclinical studies have shown that inhibitors targeting DNA methyltransferases (DNMT), histone deacetylases (HDAC), enhancer of zeste homolog 2 (EZH2), bromodomain and extra-terminal domain (BET) proteins, and lysine-specific demethylase 1 (LSD-1), can improve anti-tumor efficacy of ICB. This depends on effects of the epigenetic drugs on the tumor immune landscape, on generation of anti-tumor immunity, on modulation of immunosuppression and on immune escape processes [5–8]. However, each class of inhibitors shows specific immune-related activities. For example, DNMT inhibitors can prevent T cell exhaustion [9], activate the interferon response pathway through viral mimicry, i.e. reactivation of endogenous retroviruses [10, 11], rescue cGAS and STING gene expression [12] and improve antigen processing and presentation [13]. HDAC inhibitors, alone or in association with DNMT inhibitors, can promote T cell recruitment at the tumor site [14, 15], reprogram tumor-associated macrophages and reduce the frequency or suppressive function of Tregs and MDSCs [16, 17]. EZH2 inhibitors can rescue MHC class I transcription [18] and counteract melanoma dedifferentiation [19]. BET inhibitors can downregulate PD-L1 expression [20] and suppress expression of inflammatory genes as IL6, IFNB1, IL1B, IL12A, CXCL9 and CCL12 [21]. The emerging picture is a heterogeneous landscape of diverse and sometimes opposite immune-related activities of different classes of epigenetic drugs [7, 8]. This complexity explains why combination immunotherapy trials did not focus on a single epigenetic drug or drug class. In fact, among the > 80 recently reviewed phase I/II combination immunotherapy trials, 15 different inhibitors directed at five classes of epigenetic targets are being tested in association with nine different immune checkpoint inhibitors [5].

To shed light on the specificity of the immune-related transcriptional programs activated in tumor cells by distinct classes of epigenetic drugs, we carried out a comparative profiling of gene signatures induced by DNMT, HDAC, BET and EZH2 inhibitors in the melanoma context, a tumor type currently treated by immunotherapy. The selection of these epigenetic targets was based on an extensive literature search indicating that DNMT, HDAC, BET and EZH2 inhibitors are the most frequently tested drugs in pre-clinical models and in ongoing clinical trials of epigenetic immunomodulation in association with ICB [5–21]. We found that activation of several innate immunity pathways, a crucial step for the generation of adaptive anti-tumor responses [22] and for the efficacy of immune checkpoint blockade [23], characterized the immune-related transcriptional program specifically induced in melanoma cells by the DNMT inhibitor guadecitabine, compared to the other drugs. These results provide a preclinical rationale supporting epigenetic immunomodulation by DNMT inhibitors in combination with ICB. Analysis of tumor biopsies from patients in the Italian Network for Tumor Biotherapy (NIBIT) Phase Ib guadecitabine plus ipilimumab NIBIT-M4 trial [24] (NCT02608437) and of the TCGA melanoma dataset provided evidence for the clinical and prognostic significance of guadecitabine-specific immune-related genes and UR signatures.

## Methods

### Tumor cell lines

Melanoma cell lines (*n =* 14) were established, maintained and routinely tested for the absence of mycoplasma contamination by PCR, as previously described [25]. The origin of the cell lines from primary lesions (*n =* 1) or metastases (*n =* 13), histopathological features of the corresponding primary melanoma, cell line authentication by STR profiling (Gene-Print10 kit, Promega) and mutational profile by targeted next generation sequencing (NGS) are described in Suppl. Table S1A-D. Expression of genes targeted by epigenetic drugs in *n =* 10 melanoma cell lines (Suppl. Fig. S1) was evaluated as described in Supplementary methods. Mesothelioma cell lines (MPP-89, MM98 and MES2a) were obtained from the pleural effusion of mesothelioma patients, cultured, and treated with guadecitabine as previously described [26].

### Epigenetic drugs and treatments

Melanoma cell lines were treated with DNMT inhibitors decitabine (Selleckchem) and guadecitabine (MedChemExpress), HDAC inhibitor givinostat (ITF-2357, Selleckchem), BET inhibitors JQ-1 (Selleckchem) and OTX015 (Selleckchem), EZH2 inhibitor GSK-126 (Selleckchem), and CDK4/6 inhibitor abemaciclib (Selleckchem). Susceptibility of ten melanoma cell lines to all the drugs (dose range: 7.8 to 3000 nM) was assessed by the 3-(4,5) dimethylthiazol-2,5-diphenyltetrazolium bromide (MTT) assay at 96 h as described [27]. Drug doses for all subsequent experiments were tailored for each cell line and for each drug to achieve, in all instances, a response of > 60% of the control in the MTT assay (Suppl. Table S2A,B). For all subsequent assays, melanoma cells were seeded at 1.25 × 10^4^/mL in T75 flasks (Greiner Bio-One) with RPMI-1640 medium (Life Technologies Limited) supplemented with 4% FCS (Biological Industries) without antibiotics. Since the activity of epigenetic drugs is coupled with cell proliferation [28], melanoma cells were treated twice with each drug (at T = 24 h and T = 72 h) and then evaluated at T = 144 h.

### NGS analysis

Next generation sequencing (NGS) assays on cell line DNA were performed as described in Supplementary methods.

### RNA extraction

RNA was extracted from melanoma cell lines using the TRIzol reagent (Thermo Fisher Scientific). Melanin removal was performed by the CTAB-UREA method [29]. The samples were treated with DNase (Qiagen) and purified using the RNeasy MinElute Cleanup Kit (Qiagen). The quality of the total RNA was first assessed using RNA 6000 Pico Assay RNA chips run on an Agilent Bioanalyzer 2100 (Agilent Technologies). RNA was also extracted from tumor nodules previously obtained from immunodeficient mice treated or nor with guadecitabine and bearing human melanoma cell line 195 xenografts [30, 31]. Gene expression analysis was performed as described in Supplementary methods.

### Quantitative real-time methylation specific PCR (qMSP) analysis

Genomic DNA was extracted from 9 melanoma cell lines treated or not with guadecitabine (at 1 μM), using QIAmp DNA Blood mini-Kit (Qiagen), and modified (500 ng) with sodium bisulfite using the EZ DNA Methylation-Gold Kit (Zymo Research). Details on qMSP and primers for the analysis of the methylation status of LINE-1, MAGE-A1 and NY-ESO-1 are described in Supplementary methods.

### Quantitative Western blot

SDS-PAGE was performed with 30 μg of protein lysate on 4–12% NuPAGE Bis-Tris (Thermo Fisher Scientific), in MOPS buffer as described in Supplementary methods. The primary antibodies used are listed in Suppl. Table S3.

### Data analysis

Transcriptomic Analysis Console (TAC) software (Applied Biosystems, Thermo Fisher Scientific) and nSolver software 4.0 were used for analysis of Clariom S gene expression data and of NanoString data respectively, as described in Supplementary methods. Edwards-Venn diagrams were generated by VENNTURE software [32]. Upstream regulator and canonical pathway analyses were performed by Ingenuity Pathway Analysis (IPA 8.5, www.ingenuity.com) as described in Supplementary methods. RNA-seq data from tumor samples obtained at baseline (week 0), at week 4 and week 12 of treatment were retrieved from the published NIBIT M4 Phase Ib trial [24]. Raw FASTQ files of were aligned to the human reference genome (GRCh38/hg38) using STAR version 2.7.0b and gene expression level was quantified using featureCounts version 1.6.3. Downstream analyses were performed using the R statistical environment. The EDASeq package was used to normalize the count matrix using GC-correction for the within-normalization step and upper-quantile for the between-phase. The EdgeR package was used to identify differentially expressed genes in tumor biopsies obtained at different times points from NIBIT-M4 patients. The guadecitabine-specific immune-related gene signature, identified in this study, was used to construct a heatmap of the Log2 fold changes for the comparisons of tumor biopsies at different time points. The ClusterProfiler package was used to perform and visualize GSEA pre-ranked analyses using the guadecitabine-specific gene signature for each comparison.

## Results

### Epigenetic drugs induce different profiles of gene expression in melanoma cell lines

Ten human melanoma cell lines were characterized for the tissue of origin (Suppl. Table S1A), the mutational profile by targeted NGS (Suppl. Table S1D) and the whole genome gene expression profile by Clariom S arrays. Gene profiling data were used to check expression of epigenetic drug targets (Suppl. Fig. S1) and of differentiation-related signatures (Suppl. Fig. S2) as defined by Tsoi et al. [33]. Response of these cell lines to four epigenetic drugs targeting DNA methyltransferases (guadecitabine), HDAC (givinostat), BET proteins (JQ1 and OTX-015) and EZH2 (GSK126) was tested by the MTT assay (Suppl. Fig. S3). The CDK4/6 inhibitor abemaciclib, a drug with immunomodulatory activity unrelated to epigenetic regulation [34], was also included. Drugs as the BET, HDAC and CDK4/6 inhibitors showed dose-dependent anti-proliferative effects on melanoma cell lines (Suppl. Fig. S3). Therefore, drug doses for all subsequent experiments were tailored for each cell line and for each drug to achieve, in all instances, a response of > 60% of the control in the MTT assay (Suppl. Table S2A,B). Biological activity of guadecitabine was also checked by quantitative methylation specific PCR (qPCR) analysis in 9 melanoma cell lines. Results demonstrated a significant (*p* < 0.05) reduction in the extent of methylation of *LINE-1* repetitive elements, chosen as a surrogate of the overall genomic DNA methylation, and in the levels of specific promoter methylation of cancer testis genes (i.e., *MAGE-A1* and *NY-ESO-1*) by guadecitabine treatment (Suppl. Fig. S4).

**Fig. 1:**
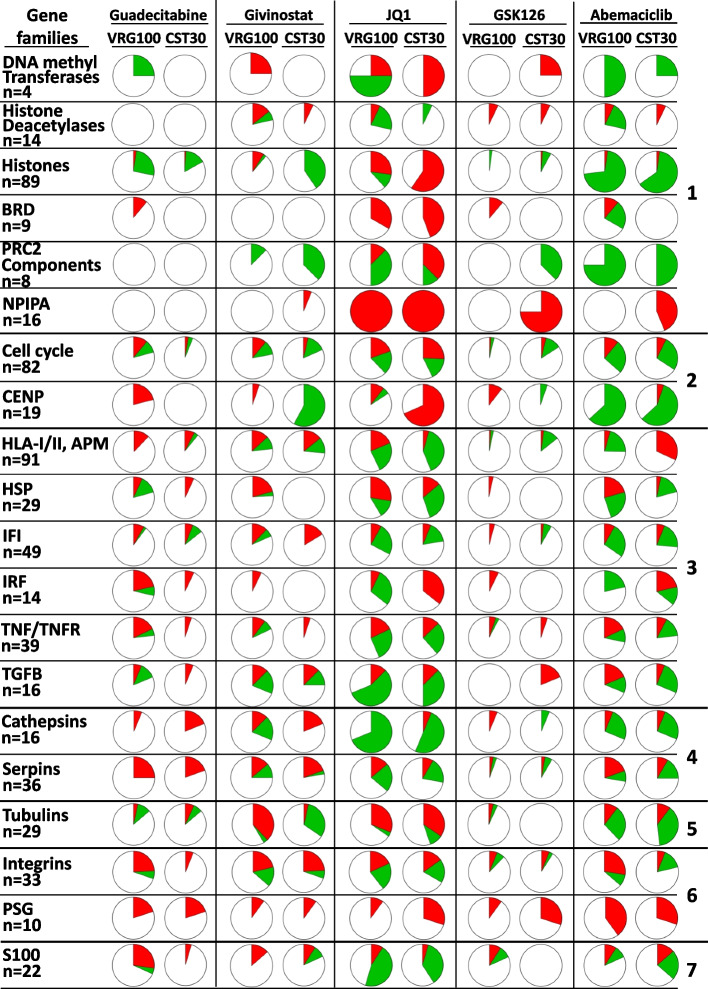
Landscape of gene modulation by epigenetic drugs in melanoma cell lines. Each pie chart shows the % of genes significantly modulated in each of 20 families or functional groups (red = upregulated genes, green = downregulated genes) by each drug in the two cell lines (VRG100 and CST30), on the basis of FC and *p* value. The 20 gene families are further classified into 7 groups identified on the right hand side of the Figure: 1, epigenetic regulation; 2, proliferation; 3, immune regulation; 4, enzymes; 5, structural components; 6, adhesion; 7, differentiation

Two of the cell lines (VRG100 and CST30) were selected for the initial gene modulation experiments assessed with Clariom S arrays. VRG100, isolated from an NRAS-mutant tumor, has a more undifferentiated/mesenchymal transcriptomic profile, as indicated by higher expression of genes as *AXL, EGFR, ZEB1, TGFBI, SPOCK1, PVRL3, CTGF* (Suppl. Fig. S5). CST30 was isolated from a BRAF-mutant tumor and is characterized by a neural crest-like/transitory differentiation program, as shown by higher expression of genes as *MITF, SOX10, PMEL, MLANA, TYR, DCT, ERBB3* (Suppl. Fig. S5). The five drugs modulated 6 to 30% of the genes in the two cell lines (Suppl. Fig. S6). Edwards-Venn diagrams [32], showed both drug-specific and shared modulatory activities (Suppl. Fig. S7). Guadecitabine, givinostat, JQ1, GSK-126 and abemaciclib exerted modulatory effects on 20 gene families grouped into seven biological functions (Fig. 1). Drugs impacted on expression of gene families related to epigenetic regulation, proliferation, immune regulation, enzymatic activity, structural function, adhesion and cellular differentiation. Variable modulatory activity was also observed on epigenetic regulator genes (DNMT, HDAC, BET and PRC2 components), and on genes encoding histones and nuclear pore complex interacting proteins (NPIPA genes). Immunomodulation emerged as a dominant feature: guadecitabine, givinostat and JQ1 were the most active drugs, followed by GSK-126, in the modulation of genes encoding HLA-I/II and antigen processing machinery (APM) components, heat shock proteins, IFN (IFI and IRF), TNF/TNFR and TGFβ pathways. These immune-related genes were frequently upregulated by guadecitabine and givinostat, but downregulated by JQ1 (Fig. 1).

### Identification of immune-related gene signatures induced by epigenetic drugs in melanoma

**Fig. 2:**
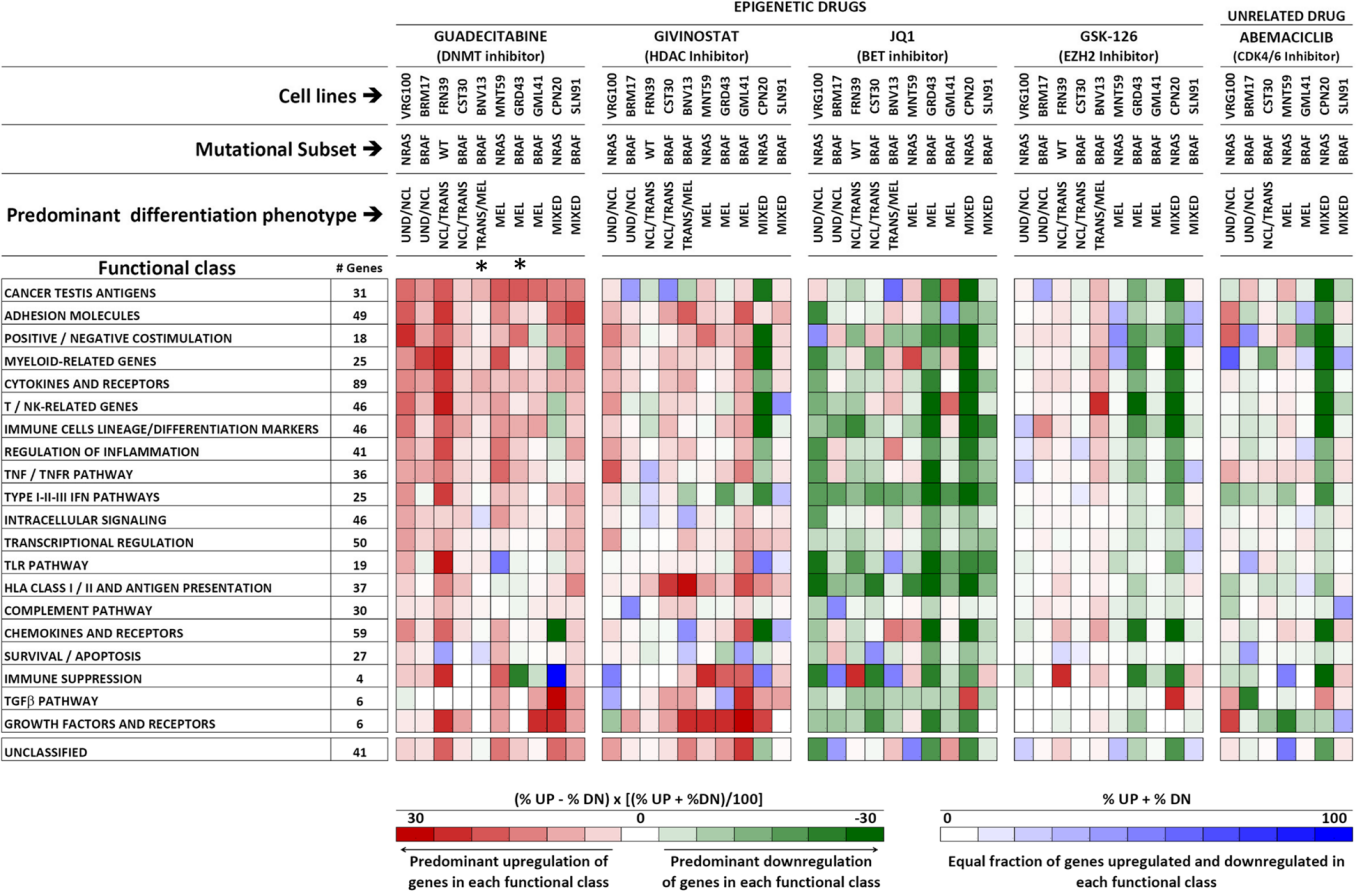
Immune-related gene modulation by epigenetic drugs in melanoma. Modulation of 731 genes, grouped into 21 functional classes, was assessed by the NanoString Cancer Immune panel upon treatment with 4 epigenetic drugs and with Abemaciclib of ten melanoma cell lines with the indicated mutational and differentiation profiles. The overall modulatory activity of all drugs on each gene class was visualized by two ad-hoc developed, color-coded metrics described at the bottom of the Figure. Up- or down-modulation of each gene by any drug was defined on the basis of a Treated/Control ratio > |1.5|. *: these two cell lines were treated with decitabine, the active metabolite of guadecitabine

We used the NanoString Cancer Immune panel, containing 731 immune-related genes re-classified into 21 functional classes (Suppl. Fig. S8), to test the modulatory effects of all inhibitors on ten melanoma cell lines. Quantitative analysis (Suppl. Fig. S9) and hierarchical clustering of NanoString data (Suppl. Fig. S10) indicated strong drug-related specificity in gene modulation. To reduce the complexity of the modulatory effects induced by the drugs on the 21 functional classes (Suppl. Table S4, sheets 1 to 21), we used two ad-hoc developed metrics (Fig. 2) allowing to visualize the proportion of gene significantly modulated in each class and the predominant direction of the effects, in terms of gene up- or down-regulation (Fig. 2). Guadecitabine and givinostat mainly induced upregulation of immune-related genes in the majority of cell lines, irrespective of mutational or differentiation profile of the cell lines (Fig. 2). The BET inhibitor JQ1 mainly suppressed immune-related gene expression (Fig. 2), while GSK126 and abemaciclib were less active and showed cell line-specific effects. Treatment of two cell lines (BNV13 and GRD43) with decitabine, the active metabolite in-vivo of guadecitabine, induced similar effects as those observed in cell lines treated with guadecitabine (Fig. 2). Guadecitabine induced frequent upregulation of genes of the following functional classes: cancer testis, adhesion molecules, positive/negative co-stimulation, myeloid-related, cytokines and receptors, T/NK- related, immune cell lineage/differentiation, regulation of inflammation, TNF/TNFR pathway, Type I-II-III IFN pathways and intracellular signaling (Fig. 2 and Suppl. Table S4, sheets 1 to 21, for gene level analysis). Guadecitabine was also active in upregulation of several anti-viral genes in the type I-II-III IFN pathways, including IFI27, IFITM1, ISG20, and IFNL2, while the BET inhibitor JQ1 invariably suppressed these genes (Suppl. Table S4, sheet 10). JQ1 induced frequent down-regulation of genes related to immune cells lineage/differentiation markers, TNF/TNFR pathway, Type I-II-III IFNs pathways, intracellular signaling and transcriptional regulation, TLR pathway, HLA Class I/II and antigen presentation pathways (Fig. 2 and Suppl. Table S4, sheets 1 to 21). Treatment of six cell lines with OTX-015, a different BET inhibitor tested in clinical trials [35], confirmed the predominant down-modulation of immune-related genes induced by JQ1 (Suppl. Fig. S11).

We then generated the epigenetic drug-specific signatures, listing genes most frequently modulated in the cell lines according to each of 21 functional classes (Suppl. Fig. S12). The guadecitabine-specific signature contained 170 genes, mostly upregulated by this DNMT inhibitor, while the JQ1-specific signature contained 217 genes, in most instances downregulated by the BET inhibitor. The givinostat-specific gene signature contained 128 genes, while the GSK126 signature contained 40 genes (Suppl. Fig. S12).

### Epigenetic drugs modulate expression of immune-related proteins in melanoma

**Fig. 3:**
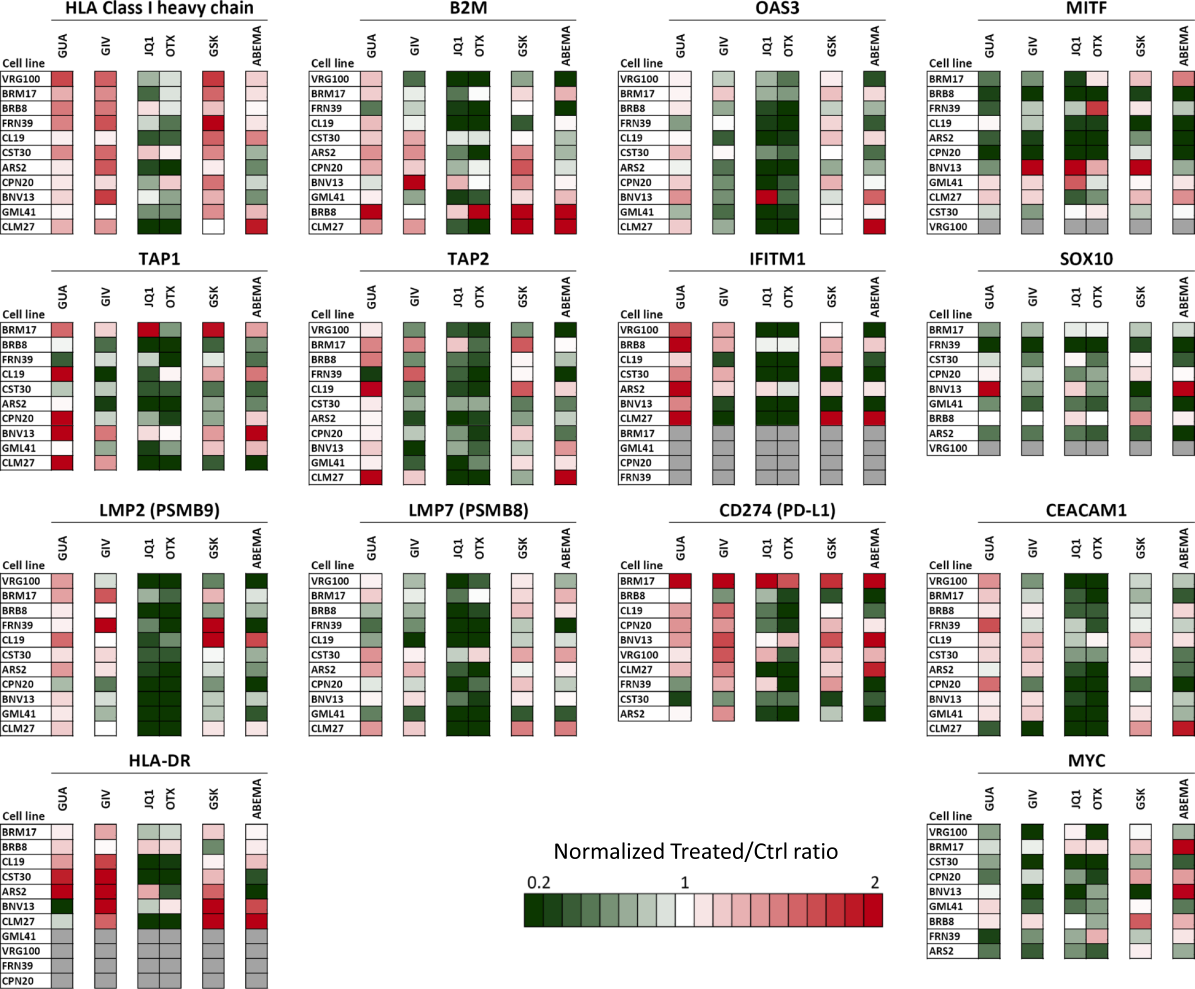
Quantitative western blot analysis for modulation of immune-related proteins in melanoma cell lines by epigenetic drugs. Eleven melanoma cell lines were treated with guadecitabine (GUA), givinostat (GIV), JQ1, OTX-015 (OTX), GSK-126 (GSK) and abemaciclib (ABEMA) and assessed for expression/modulation of 14 proteins. Data analysis and visualization approach described in Suppl. Fig. S12 and S13. Grey boxes: the antigen was not expressed and not induced by treatments

Quantitative western blot (Suppl. Fig. S13 and S14 for details on data analysis) was used to test epigenetic drug-induced modulation of 14 immune-related and differentiation-related proteins in 11 melanoma cell lines. Results confirmed the findings observed at the gene level. Guadecitabine, followed by givinostat and GSK-126, was the most active drug in upregulation of HLA class I and II-related proteins (HLA Class I heavy chain, B2M and HLA-DR). In the panel, six cell lines were constitutively HLA-DR-positive (BRM17, CST30, BNV13, BRB8, CL19, ARS2, CLM27) and these molecules could be often upregulated by guadecitabine, givinostat and GSK126, while mostly downregulated by the BET inhibitors JQ1 and OTX-015. Four cell lines were found to be constitutively negative (GLM41, VRG100, FRN39, CPN20) and epigenetic drugs could not induce expression. Guadecitabine was more active compared to givinostat and GSK-126 on molecules (TAP1, TAP2, LMP2/PSMB9) belonging to the antigen processing and presentation pathway (Fig. 3). The two BET inhibitors induced, in most instances, a reduction of expression of these classes of proteins. Guadecitabine and BET inhibitors showed opposite effects on the expression of IFN-pathway related proteins (OAS3 and IFITM1), on PD-L1 / CD274 and CEACAM1. All epigenetic drugs had a predominant inhibitory effect on the expression of the melanoma differentiation-related proteins MITF and SOX-10 and of the transcription factor MYC (Fig. 3).

### Combinatorial treatments with epigenetic drugs indicate a dominant effect of guadecitabine and JQ1 on immune-related gene modulation

Melanoma cell lines from the panel were treated with six binary combinations of the four epigenetic drugs (guadecitabine + givinostat, guadecitabine + JQ1, guadecitabine + GSK126, givinostat + JQ1, givinostat + GSK126, JQ1 + GSK126). Gene modulation was tested by the NanoString Cancer Immune panel and compared to the effects of single drugs (Suppl. Table S5, sheets 1 to 5). In most instances, combinatorial treatments did not enhance gene modulation compared with single treatments. Moreover, the association of guadecitabine with givinostat or GSK-126 replicated the pattern of immune-related gene upregulation induced by guadecitabine alone (Suppl. Table S5, sheet 1). Similarly, any association of JQ1 with other drugs replicated the dominant suppressive effect on immune-related gene expression exerted by JQ1 alone (Suppl. Table S5, sheet 4). These results argue against the possibility of achieving a more effective modulation of immune-related genes by combining two epigenetic drugs belonging to different classes.

### Identification of upstream regulator signatures and functional networks regulated by epigenetic drugs

To gain insight into the transcriptional networks and biological pathways regulated by the different epigenetic agents we used upstream regulator (UR) analysis, with IPA (Suppl. Fig. S15 for pipeline of data analysis). This computational tool identifies upstream transcriptional regulators that control downstream genes, explains the observed gene expression changes in the dataset and predicts the functional status (activated or inhibited) of each UR molecule. Each of the predicted UR can be associated with the main cellular processes and pathways it belongs to allow an improved understanding of the biological activity of each drug (Suppl. Fig. S15).

**Fig. 4:**
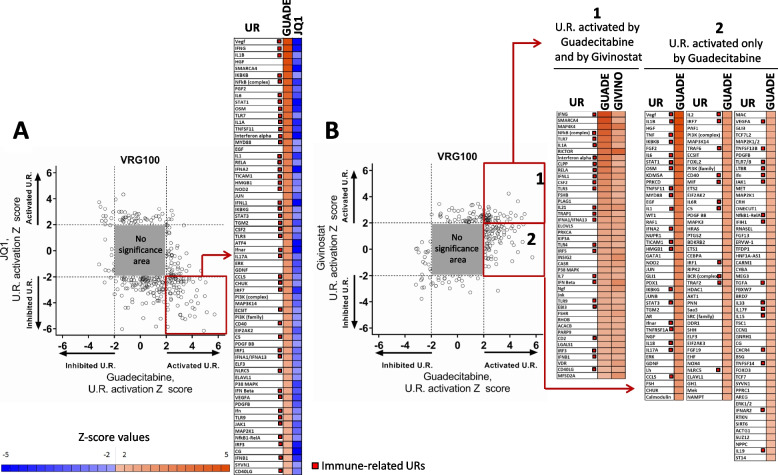
Comparison of URs activated or inhibited by guadecitabine vs JQ1 and by guadecitabine vs givinostat in melanoma cell line VRG100. **A**. Scatter plot of significantly modulated URs by Guadecitabine and JQ1. The grey square represents the area of no-significance, i.e. URs with Z < |2| and p value > 0.05. URs in the red square, representing factors showing activation by guadecitabine and inhibition by JQ1, are listed in the table on right of the scatter plot. **B**. Scatter plot of significantly modulated URs by guadecitabine and givinostat. URs in red square 1 represent factors showing activation by both guadecitabine and givinostat; URs in red square 2 represent factors activated only by guadecitabine and not by givinostat. URs in square 1 and 2 are listed in the tables on the right side of the scatter plot

UR analysis, based on *p* values and activation Z scores (Suppl. Table S6), was initially applied to the whole genome gene expression dataset of cell lines VRG100 and CST30, providing evidence for a wide range of biological activities of all drugs. Several of the significant URs, involved in immune regulation (highlighted in green in Suppl. Table S6), were often predicted to be activated by guadecitabine, and, to a lesser extent by givinostat, but were frequently inhibited by JQ1 or GSK126. URs significantly modulated by at least two different inhibitors were grouped according to 18 functional groups (Suppl. Fig. S16 and S17) revealing the amplitude, specificity and frequent diversity of the biological pathways affected by the epigenetic drugs. For example, guadecitabine exerted opposite effects compared to JQ1 on UR molecules related to angiogenesis, epigenetic regulation, growth factors and receptors, and intracellular signal transduction (Suppl. Fig. S16 and S17). A large set of these URs (Group 12 and Group 14 respectively in Suppl. Fig. S16 and S17) were immunity/inflammation-related molecules belonging to at least seven functional groups/pathways (cytokines, IFNs, JAK-STAT, IRFs, TLR, NF-kB, TNF) frequently activated by guadecitabine, and to a lesser extent by givinostat, while almost invariably predicted to be inhibited by JQ1. Scatter plots of URs significantly modulated by guadecitabine vs. JQ1, in the cell line VRG100 (Fig. 4 A), confirmed the opposite biological activity of these two drugs. A large number of immune-related URs activated (i.e. z score > 2) by guadecitabine (highlighted in the red box and listed in the accompanying panel, Fig. 4A) were instead inhibited (i.e. z score < − 2) by JQ1. Scatter plots comparing the URs modulated by guadecitabine and givinostat revealed a set of URs activated by both drugs (highlighted in red box #1 and listed in the accompanying panel, Fig. 4B). Moreover, URs activated only by guadecitabine but not by givinostat were identified (highlighted in red box #2 and listed in the accompanying panel, Fig. 4B).

The two scatter plots (Fig. 4A,B) also indicated that there were additional URs inhibited by guadecitabine, or givinostat or JQ1. The molecular identity of these URs is shown in Supplementary Table 6.

The most significant URs predicted to be activated or inhibited by each drug were used to generate, by IPA, a graphical abstract summarizing, as functional networks, the biological activity in melanoma of the epigenetic inhibitors (Suppl. Fig. S18). Guadecitabine biological activity was mainly characterized by activatory relationships linking several master regulators of innate immunity (IFN-γ, TNF-α, TLR2, TLR7, MYD88, IL1A, IL1B, IL-6), of the beta catenin pathway (WNT3A, CTNNB1) and of the RNA recognition pathway (MAVS, DDX58/RIG-1, EIF2AK2). These networks converged on intracellular pathways controlling inflammatory gene expression (MAPK14/p38 MAPK, FOXO1, TGM2). JQ1 was characterized by induction of inhibitory relationship linking regulators of inflammation (IFNG, IL17A, TGFB2), melanoma biological behavior (EGFR, WNT3A, TEADs, MRTFB), cell-cell interaction (CD44, CCN2), YAP pathway (LLGL2, NSUN6) and main transcriptional regulators (FOS, JUN, FOXM1). Givinostat functional networks were characterized by activatory relationships linking the master regulator of hypoxia response (HIF1-α) to EDN1 and INSIG2 and by activation of the MRTF-SRF-MYOCD pathway. GSK126 activity was instead characterized by activatory relationship linking the beta catenin and YAP pathways to TEADs transcription factors and to secreted BMP7.

### Guadecitabine-specific UR signature molecules are activated in cell lines isolated from melanoma and other tumor types

**Fig. 5:**
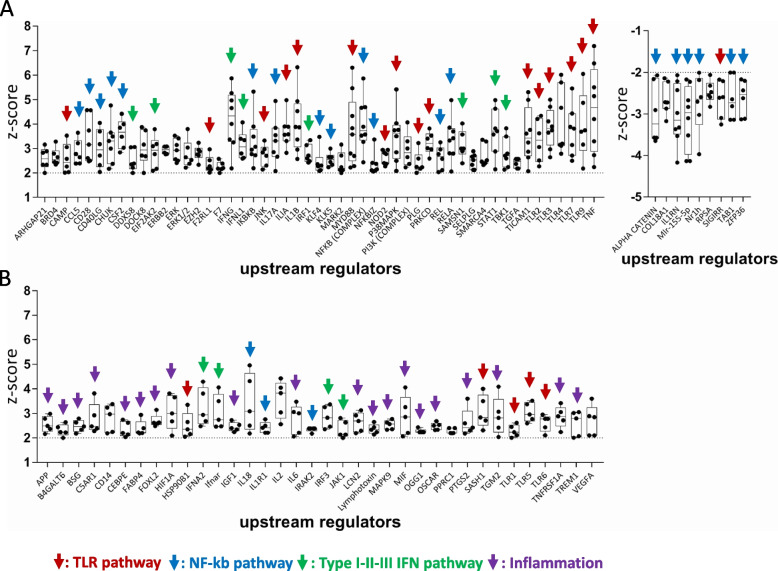
Main URs explaining the immunomodulatory function of guadecitabine. **A.** Upstream Regulators predicted to be activated (Z score > 2, p value < 0.05, left graph) or inhibited (Z score < − 2, p value < 0.05, right graph), in at least 6/10 cell lines. **B**. Upstream Regulators predicted to be activated (Z score > 2, p value < 0.05) in up to 5/10 cell lines. Data in A and B based on IPA analysis of NanoString gene modulation data. Color-coded arrows define the biological function/pathway of each UR. Each black dot in the two graphs represents the Z score value of the indicated URs, computed for a single cell line

To strengthen the characterization of immune-related URs, beyond the VRG100 and CST30 cell lines context, we extended such analysis to the whole panel of 10 melanoma cell lines screened by NanoString Cancer Immune panel upon treatment with guadecitabine. We identified 51 activated URs (Z score > 2) and 9 inhibited URs (Z score < − 2) in at least 6 out of 10 cell lines, i.e. in more than 50% of cell lines (Fig. 5A). Additional 36 inflammation-related URs were predicted to be activated in 5 out of 10 cell lines, i.e. in 50% of the cell lines (Fig. 5B). The URs were labeled with color-coded arrows pointing to the pathways/biological processes to which these molecules belong. This analysis revealed the main immune-related activity of guadecitabine as an activator of innate immunity (NF-kB, TLR and Type I-III IFN) and inflammation-related pathways (Fig. 5A,B). Several type I/III IFN, NF-kB and TLR pathways-related UR molecules, predicted to be activated by guadecitabine in-vitro in melanoma cell lines, were also predicted to be activated in-vivo by this DNMT inhibitor (Suppl. Fig. S19A), in human melanoma xenografts from a previously described immunodeficient mouse model [30, 31]. In the tumor xenografts, upregulation of specific genes belonging to the IFN-α/β and IFN-γ canonical pathways, by guadecitabine, was also observed (Suppl. Fig. S19B). Activation of several of the immune-related URs identified in guadecitabine-treated melanoma cell lines was also documented in mesothelioma cell lines treated with this DNMT inhibitor (Suppl. Fig. S20A). Similar evidence was obtained by retrieval of published gene modulation data [36] in hepatocarcinoma cell lines (Suppl. Fig. S20B). Taken together, these results indicated that the biological activity of guadecitabine, as an immunomodulatory agent, can be consistently described by its UR signature in different tumor types.

### Clinical relevance of the guadecitabine-specific gene and UR signatures in the context of the NIBIT-M4 epigenetic immunomodulation trial

**Fig. 6:**
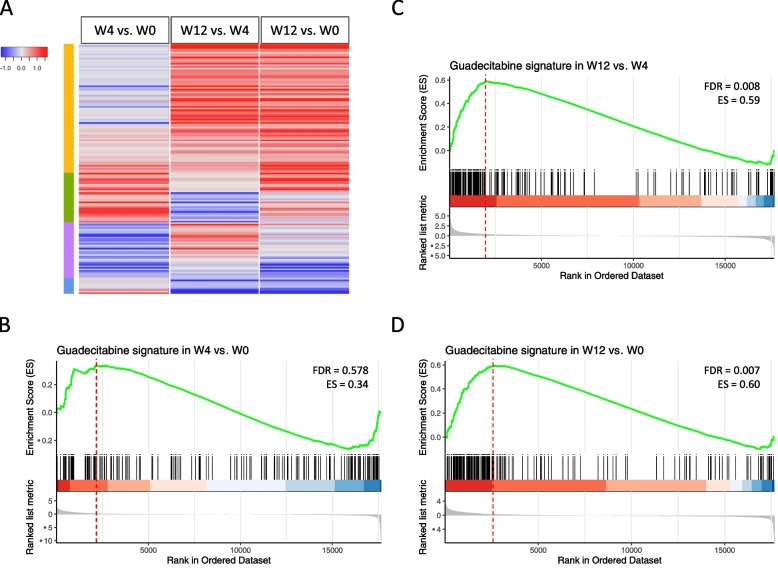
Expression and modulation of guadecitabine-specific signature genes in baseline and on-treatment clinical samples from NIBIT-M4 patients. **A**. Heatmap of log2 fold changes of guadecitabine signature genes among the following comparisons: w4 vs. w0, w12 vs. w4 and w12 vs w0. **B-D**. Enrichment plots containing profiles of the running enrichment scores (ES) and positions of guadecitabine gene set members on the rank ordered list in GSEA for w4 vs. w0 (B), w12 vs. w4 (C) and w12 vs. w0 (D) comparisons

The NIBIT-M4 trial is the first completed clinical study testing the association of guadecitabine with ipilimumab in metastatic melanoma [24]. To test the modulation of guadecitabine-specific signature genes in tumor samples from this trial we retrieved RNA-seq data from pre-therapy (week 0) and on treatment (week 4 and week 12) melanoma biopsies. Guadecitabine-specific signature genes (Fig. 6A) showed increased expression in on-treatment samples at w12 and w4 compared with baseline biopsies, and in w12 lesions compared with w4 biopsies. Almost 92% of the guadecitabine-specific signature genes were upregulated in biopsies obtained at w12 and w4, compared to w0 (Fig. 6A). By gene set enrichment analysis [37] the guadecitabine-specific gene signature showed a significant increase at w12 compared to w4 and w0, but not at w4 compared to w0 (Fig. 6B-D). We then checked two published datasets [38, 39] listing genes upregulated in on-treatment lesions compared to baseline samples of patients treated with ipilimumab in monotherapy. Among 376 and 286 significantly modulated genes in the two datasets, only 35/166 (21.1%) and 28/166 genes (16.9%) of the guadecitabine signature were upregulated in on-treatment lesions, compared to baseline (data not shown).

**Fig. 7:**
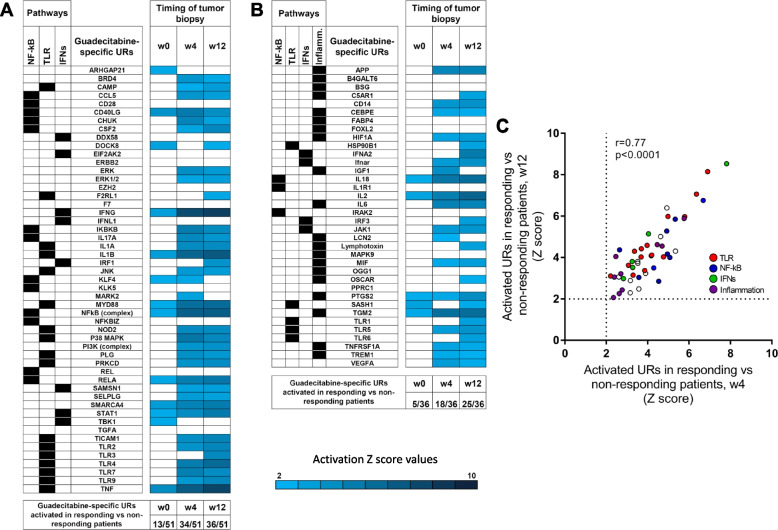
Activation of guadecitabine-specific UR signature molecules in on-treatment tumor biopsies from responding compared to non-responding patients in the NIBIT-M4 trial. **A, B**. Z score values of guadecitabine-activated URs as identified in Fig. 5A (A) or in Fig. 5B (B) based on differential gene expression analysis in tumor samples (obtained at w0, w4 and w12) from responding vs non-responding patients. Only significant URs (Z score > 2) are shown by the indicated color code. **C**. Scatter plot of Z score values of activated URs in responding vs non-responding patients at w4 vs. wk12

We then tested the hypothesis that the guadecitabine-specific UR signature molecules, identified as activated in-vitro in 6/10 or in 5/10 melanoma cell lines, could also be differentially activated in-vivo in on-treatment lesions from responding compared to non-responding NIBIT-M4 patients. To this end, we first run differential gene expression analysis in tumor biopsies in responding vs non-responding patients at each time point (w0, w4 and w12). Subsequently we used such differentially expressed genes for UR analysis by IPA. The results revealed progressive activation of several guadecitabine-specific UR signature molecules in on-treatment (w4 and w12) tumor biopsies from responding patients compared to non-responding patients (Fig. 7A,B). Three URs (ARHGAP21, KLF4, TBK1), among those that discriminated responding and non responding patients in the baseline tumor samples, lost significance in subsequent biopsies, possible reflecting the shift in the transcriptomic programs of the neoplastic lesions occurring as result of therapy. Moreover, the activated URs in w4 and w12 tumor samples showed significant correlation of the z-score values (Fig. 7C).

Taken together, these results strongly support the notion that genes in the guadecitabine-specific signature are modulated even in-vivo at tumor site in patients treated with this DNMT inhibitor. Moreover, the progressive activation of the UR signature in responding versus non-responding patients suggests that the clinical activity of guadecitabine in melanoma patients depends on the effective activation of innate immunity pathways.

### Prognostic significance of genes in the guadecitabine signature in tumors from the TCGA cancer genome atlas

We investigated the potential prognostic significance of genes belonging to the epigenetic drug-specific signatures. To this end we focused on the guadecitabine and JQ1 signatures and tested their prognostic significance in 41 tumor types of the TCGA cancer genome atlas using the outcome module of the TIMER2.0 web server (http://timer.comp-genomics.org). Sixty-five % of the genes in the guadecitabine signature were significant in a Cox proportional hazards model (Suppl. Table S7) and most of the prognostically significant genes in the signature conferred a reduced risk (z score < 0, *p* < 0.05). In the JQ1 signature, 59% of genes were significant and conferred a reduced risk (z score < 0, p < 0.05). However, the prognostically significant genes in the guadecitabine signature were upregulated by this DNMT inhibitor in melanoma cells, while the prognostically significant genes in the JQ1 signature were frequently downregulated by the BET inhibitor in melanoma cells (Suppl. Table S7). Interestingly, guadecitabine signature genes showed prognostic significance and conferred a reduced risk mainly in the melanoma TCGA dataset and not across all TCGA tumor types (Suppl. Fig. S21 for representative results on three functional classes of genes). In several other tumor types the same genes either lacked prognostic significance or conferred increased risk (Suppl. Fig. S21). Taken together, these results suggest that a large fraction of immune-related genes upregulated by guadecitabine may exert a relevant anti-tumor function in melanoma that impacts on clinical outcome.

## Discussion

Innate immunity pathways play a crucial role in anti-tumor responses, even in the context of immunotherapy [22, 23]. Therefore, immunomodulatory agents that may promote activation of innate immunity pathways may be relevant candidates for combination with immune checkpoint blockade. The results of this study indicated that the DNMT inhibitor guadecitabine may indeed represent a promising class of immunomodulatory agents, among those investigated, as this drug promoted frequent upregulation of genes belonging to several innate immunity pathways. By looking at gene expression data from the NIBIT-M4 trial we found that most of the immune-related genes in the guadecitabine-specific signature were upregulated in on-treatment tumor biopsies, but not in tumor samples from patients receiving only ipilimumab. Moreover, guadecitabine-specific UR molecules activated in on-treatment lesions discriminated responding from non-responding NIBIT-M4 patients, indicating that effective activation of innate immunity pathways in-vivo, by guadecitabine, is requested for the clinical efficacy of this DNMT inhibitor. These results also raise the possibility that lack of clinical benefit in the NIBIT-M4 trial may be due to mechanisms of resistance to DNMT inhibitors, as described in acute myelogenous leukemia patients [40]. Our study expands previous evidence on the immunoregulatory and anti-tumor activity of demethylating agents, including guadecitabine, when used either alone or in combination with anti-CTLA-4 in pre-clinical immunocompetent mouse models [30, 31, 41, 42]. Moreover, recently published early phase clinical trials testing the association of this DNMT inhibitor with the anti-PD-1 mAb pembrolizumab [43, 44] have documented significant anti-tumor and immunomodulatory effects. Taken together, the results of this study and the available pre-clinical and clinical evidence strongly supports the development of combinatorial immune intervention where DNMT inhibitors as guadecitabine are associated with ICB.

Genes in the guadecitabine-specific signature showed a prognostic significance and conferred a reduced risk in the melanoma TCGA dataset, suggesting that these immune-related genes are playing a significant role in protective immune responses against melanoma. Among genes upregulated by guadecitabine we found CTLA-4 and, interestingly, low levels of CTLA-4 promoter methylation have been associated with response to ICB [45]. In contrast to the DNMT inhibitor, JQ1 behaved mainly as an anti-inflammatory drug, in agreement with the ability of BET inhibitors to suppress inflammatory gene expression [21]. JQ1 predominantly downregulated immuno-related genes that were instead upregulated by guadecitabine and inhibited upstream regulators predicted to be activated by guadecitabine, including molecules belonging to the IFN-gamma, TLR and NF-kb pathways, targeted by this BET inhibitor [46].

The four drugs exerted modulatory effects on the expression of genes encoding DNMT, HDAC, histones, BET, and PRC2 components, suggesting that activity of these agents depends not only on inhibition of their specific targets, but also on modulation of additional genes contributing to epigenetic regulation. The drugs also affected the expression of genes encoding proteolytic enzymes (cathepsins), and their inhibitors (serpins), implicating epigenetic drugs in the regulation of different cancer-related processes, controlled by cathepsins, such as proliferation, angiogenesis, metastasis and invasion. These results extend previous knowledge on the non-immuno-related anti-tumor activities of epigenetic inhibitors, such as reactivation of tumor suppressor genes by demethylating agents [47], promotion of cell death and suppression of angiogenesis by HDAC inhibitors [48], inhibition of proto-oncogenes MYC and BCL2 by BET inhibitors [49] and downregulation of DNA repair genes by EZH2 inhibitors [50].

Taken together, our study indicates that distinct epigenetic drugs may elicit specific immune-related transcriptional programs in melanoma cells. These results provide a pre-clinical rationale in favor of association of ICB with DNMT or HDAC inhibitors, rather than with BET or EZH2 inhibitors.

## Conclusions

The findings of this study support a mechanistic rationale for development of combinatorial immune intervention where boosting of NF-kB, TLR, Type I-III IFN and inflammation-related pathways by DNMT inhibitors as guadecitabine might cooperate with the rescue of adaptive immunity by immune checkpoint blockade.

## Supplementary Information

**Additional file 1.** Supplementary Methods.

**Additional file 2: Supplementary Figs. S1 to S21. Supplementary Fig. S1.** Expression in ten melanoma cell lines of genes and gene families targeted by epigenetic drugs. **Supplementary Fig. S2.** Differentiation profile of melanoma cell lines. **Supplementary Fig. S3**. Susceptibility of ten melanoma cell lines to the anti-proliferative effects of epigenetic drugs. **Supplementary Fig. S4.** Methylation status of LINE-1, MAGE-A1 and NY-ESO-1 in 9 melanoma cell lines treated with guadecitabine. **Supplementary Fig. S5**. Volcano plot of differentially expressed genes in VRG100 and CST30 cell lines. **Supplementary FigureS6**. Whole genome gene modulation analysis by 4 epigenetic drugs in two melanoma cell lines. **Supplementary Fig. S7**. Edwards-VENN diagram analysis of significantly modulated genes by epigenetic drugs. **Supplementary Fig. S8.** Original and revised gene classification of the NanoString nCounter PanCancer Immune Profiling panel. **Supplementary Fig. S9**. Quantitative analysis of Nanostring data in ten melanoma cell lines treated with epigenetic drugs. **Supplementary Fig. S10**. Modulation of immune-related genes in melanoma cell lines by epigenetic drugs. **Supplementary Fig. S11**. Comparison of immune-related gene modulation by BET inhibitors JQ1 and OTX-015. **Supplementary Fig. S12**. Immune-related signature of epigenetic drugs in melanoma. **Supplementary Fig. S13**. Outline of the strategy for quantitative analysis and visualization of western blot data. **Supplementary Fig. S14**. Quantitative western blot analysis and visualization of the modulation of LMP7 by epigenetic drugs in 11 melanoma cell lines. **Supplementary Fig. S15.** Pipeline of data analysis based on Upstream Regulators (UR) identified by IPA. **Supplementary Fig. S16.** Classification of URs significantly modulated by at least two different drugs in melanoma cell line VRG100. **Supplementary Fig. S17**. Classification of URs significantly modulated by at least two different drugs in melanoma cell line CST30. **Supplementary Fig. S18.** Summary of the major functional networks linking relevant URs emerging from IPA Core Analysis in melanoma cell line VRG100. **Supplementary Fig. S19.** Comparison of URs activated by guadecitabine in-vitro and in-vivo. **Supplementary Fig. S20**. Comparison of URs activated by guadecitabine in melanoma cell line VRG100 vs a mesothelioma cell line vs hepatocarcinoma cell lines. **Supplementary Fig. S21.** Prognostic significance of selected genes in the guadecitabine signatures in 41 TCGA tumor types.

**Additional file 3 Supplementary Table S1.** Origin of melanoma cell lines, histopathological features of the corresponding primary tumor, cell line authentication data by STR profiling, and mutational profile by NGS of 14 melanoma cell lines used in this study.

**Additional file 4: Supplementary Table S2.** Drug doses used for experiments and effect of each drug at such dose in the MTT assay on each cell line.

**Additional file 5: Supplementary Table S3.** Antibodies used in quantitative western blot analysis.

**Additional file 6: Supplementary Table S4.** Modulation of immune-related genes belonging to 21 functional classes by epigenetic drugs. Sheets # to #21.

**Additional file 7: Supplementary Table S5.** Immune-related gene modulation by epigenetic drugs and by combinatorial treatments.

**Additional file 8: Supplementary Table S6.** Upstream Regulator analysis on differentially expressed genes in drug-treated vs untreated cell lines VRG100 and CST30.

**Additional file 9 Supplementary Table S7.** Prognostic significance of genes in the guadecitabine and JQ1 immune-related signatures in the Cancer Genome Atlas (TCGA) melanoma dataset.

## Data Availability

The datasets supporting the conclusions of this article are available in the Gene Expression Omnibus (GEO) repository with the following GEO accession numbers: GSE189628, GSE189629, GSE189630, and GSE189631. The data analyzed during this study concerning gene expression in tumor samples from patients enrolled in the published NIBIT-M4 trial were retrieved from ref. 24.

## Abbreviations

BET: Bromodomain and extra-terminal domain proteins
DNMT: DNA methyltransferases
EZH2: Enhancer of zeste homolog 2
HDAC: Histone deacetylases
ICB: Immune checkpoint blockade
IPA: Ingenuity Pathway Analysis
LSD-1: Lysine-specific demethylase 1
MTT: 3-(4,5) dimethylthiazol-2,5-diphenyltetrazolium bromide
NGS: Next generation sequencing
NIBIT: Italian Network for Tumor Biotherapy
NPIPA: Nuclear pore complex interacting proteins
TAC: Transcriptomic Analysis Console
UR: Upstream regulator
