# Supplementary material for: Landscape of immune-related signatures induced by targeting of different epigenetic regulators in melanoma: implications for immunotherapy
Source: J Exp Clin Cancer Res. 2022 Nov 17;41:325. doi: 10.1186/s13046-022-02529-5 (PMC9670381; doi:10.1186/s13046-022-02529-5)
Supplement: Supplementary file 1 — Additional file 1. Supplementary Methods. [file 13046_2022_2529_MOESM1_ESM.pdf]

Supplementary Methods

Landscape of immune-related signatures induced by targeting of different epigenetic regulators in melanoma: implications for immunotherapy.

Andrea Anichini[1], Alessandra Molla[1], Gabriella Nicolini[1], Valentina E. Perotti[1], Francesco Sgambelluri[1], Alessia Covre[2,3], Carolina Fazio[2], Maria Fortunata Lofiego[2], Anna Maria di Giacomo[2,3], Sandra Coral[2], Antonella Manca[4], Maria Cristina Sini[4], Marina Pisano[4], Teresa Noviello[5,6], Francesca Caruso[5,6], Silvia Brich[7], Giancarlo Pruneri[7,8], Andrea Maurichi[9], Mario Santinami[9], Michele Ceccarelli[5,6], Giuseppe Palmieri[4,10], Michele Maio[2,3*], Roberta Mortarini[1*]

On behalf of the **EP**igenetic **I**mmune-oncology **C**onsortium **A**IRC (**EPICA**) investigators.

[1]Human Tumors Immunobiology Unit, Dept. of Research, [7]Department of Pathology and Laboratory Medicine, [9]Melanoma and Sarcoma Unit, Department of Surgery, Fondazione IRCCS Istituto Nazionale dei Tumori, Milan, Italy. [2]Center for Immuno-Oncology, University Hospital of Siena, Siena, Italy. [3]University of Siena, Siena, Italy. [4]Unit of Cancer Genetics, National Research Council (CNR), Sassari, Italy. [10]University of Sassari, Sassari, Italy. [5]Department of Electrical Engineering and Information Technology (DIETI), University of Naples "Federico II", Naples, Italy. [6]BIOGEM Institute of Molecular Biology and Genetics, Ariano Irpino, Italy. [8]University of Milan, School of Medicine, Italy.
*These authors contributed equally.

Correspondence: Andrea Anichini, PhD, Human Tumors Immunobiology Unit, Dept. of Research, Fondazione IRCCS Istituto Nazionale dei Tumori, Via Venezian 1, 20133 Milan, Italy.
Phone+390223902817. e-mail: andrea.anichini@istitutotumori.mi.it.

Supplementary Methods.

NGS analysis. DNA was extracted from 0.5-1x10^6 melanoma cell lines using the Purelink Genomic DNA Mini Kit (Invitrogen). RNAse treatment was performed using RNAse cocktail (Invitrogen). Quantitative assessment of DNA was performed using a Nanodrop 2000 spectrophotometer and DNA integrity was confirmed by gel electrophoresis. NGS assays on melanoma cell lines DNA were performed using Ion GeneStudio S5 System and carried out on Ion AmpliSeq™ Comprehensive Cancer Panel, which provides highly multiplexed target selection of 409 genes implicated in cancer pathogenesis. Starting DNA and libraries were accurately quantified using a fluorescence-based method, such as Qubit dsDNA HS. Data analysis workflow was performed by automated data

transfer, from the Ion Torrent™ Server to the Ion Reporter Server for variant analysis; it includes result filtering, annotation, and data analysis results. To get a total amount of at least 10 mutated alleles for each candidate amplicon, the following mutation selection criteria were adopted: coverage of >200 reads and frequency of mutated alleles >5% for gene amplicon. The copy number variation (CNV) determination was obtained by adding a custom control copy number baseline to the comprehensive cancer profile analysis workflow. Results of NGS analysis of 14 melanoma cell lines used in this study are shown in Supplementary Table S1D**.**

Gene expression analysis. The total RNA (20 ng to 50 ng) was reverse transcribed using GeneChip® WT Pico Reagent Kit (Affymetrix; Thermo Fisher Scientific, Inc.). The resulting cDNA was used as a template for in vitro transcription using the same kit. The obtained antisense cRNA was purified using Nucleic Acid Binding Beads (GeneChip® WT Pico Reagent Kit, Affymetrix) and used as a template for reverse transcription to produce single-stranded DNA in the sense orientation. During this step, dUTP was incorporated. The DNA was then fragmented using uracyl DNA glycosylase (UDG) and apurinic/apyrimidinic endonuclease 1 (APE 1) and labeled with DNA reagent covalently linked to biotin using terminal deoxynucleotidyl transferase (TdT, GeneChip® WT Pico Reagent Kit, Affymetrix). Hybridization of each fragmented and labeled target was performed using the GeneChip® Hybridization, Wash and Stain Kit (Affymetrix; Thermo Fisher Scientific, Inc). A single GeneChip® Human Clariom S was then hybridized with each biotin-labeled sense target. GeneChip arrays were scanned using an Affymetrix GeneChip® Scanner 3000 7G using default parameters. Affymetrix GeneChip® Command Console software (AGCC) was used to acquire GeneChip® images and generate .DAT and .CEL files. Gene expression data were analyzed by Transcriptomic Analysis Console (TAC) software (Applied Biosystems, Thermo Fisher Scientific). Modulation of immune-related genes by epigenetic drugs in ten melanoma cells was assessed by the NanoString nCounter PanCancer Immune profiling panel enabling determination of 731 genes

(NanoString Technologies, Seattle, USA). The manufacturer's gene classification associated with the PanCancer Immune profiling panel was revised by retrieving information on gene function at http://genecards.org and then by grouping genes into 21 functional classes. For NanoString experiments panel probes (capture and report) and 200 ng of RNA were hybridized overnight at 65 °C for 16 h. Samples were scanned at maximum scan resolution capabilities (555 FOV) using the nCounter Digital Analyzer. Quality control of samples, data normalization and data analysis were performed using nSolver software 4.0 (NanoString Technologies).

Whole gene expression profile of treated and untreated mesothelioma cell lines was performed by Agilent whole human genome oligo microarray kits. The quantity and the quality of RNA, extracted as previous described, was assessed with NanoDrop® ND-1000 UV-Vis Spectrophotometer (NanoDrop Technologies, Wilmington, DE, USA) and the Agilent 2100 Bioanalyzer (Agilent Technologies, Santa Clara, CA, USA). *In vitro* transcription, labeling and purification of dye-labeled cRNA were performed using the Quick Amp Labeling Kit, one-color (Agilent Technologies, Santa Clara, CA, USA) following manufacturer's guidelines. Gene expression profiling was performed by a One-Color strategy using Cy3-labeled aRNA from guadecitabine-treated and untreated cells (Quick Amp Labeling, Agilent Technologies, Santa Clara, CA, USA). A mixture of 1650 ng of Cy3-labeled reference cRNA, Blocking Agent and Fragmentation Agent was hybridized to Whole Human Genome (1x44K) oligo microarray platform (Agilent Technologies, Santa Clara, CA, USA). Hybridization was performed for 17 hours at 65°C in 2x GEx Hybridation Buffer HI-RPM (Agilent Technologies, Santa Clara, CA, USA), using Agilent's Hybridization Oven at 10 rpm. Following washing, slides were analyzed by Agilent Microarray Scanner. Feature Extraction Software provided by Agilent (version 9.5.3) was used to quantify the intensity of fluorescence images and to normalize results by subtracting local background fluorescence

according to the manufacturer’s instruction. Genes modulated with a FC ≥ 2 or ≤ -2 in treated vs untreated cells were used for upstream regulator analyses.

Quantitative Real-Time Methylation Specific PCR (qMSP) Analysis. Genomic DNA was extracted from 9 melanoma cell lines untreated and treated with 1 µM of guadecitabine, using QIAmp DNA Blood mini-Kit (Qiagen, Hilden, Germany), and modified (500 ng) with sodium bisulfite using the EZ DNA Methylation-Gold Kit (Zymo Research, CA, United States). SYBR green qMSP reactions were performed with methylated- or unmethylated-specific primer pairs on 2 µl of bisulfite-modified genomic DNA. Primers for the analysis of the methylation status of LINE-1, MAGE-A1 and NY-ESO-1 were designed using the free on-line software MethPrimer, and are reported in the following table.

		Forward	*Reverse*
LINE-1	*Unmetilated*	TGTGTGTGAGTTGAAGTAGGGT	ACCCAATTTTCCAAATACAACCATCA
	Methylated	CGCGAGTCGAAGTAGGGC	ACCCGATTTTCCAAATACGACCG
MAGE-A1	*Unmetilated*	GTTAGGTTTTTTGGTTGAATTTTAT	CCCAAATATAACACCACTAACTTACA
	Methylated	TAGGTTTTTTCGGTCGAATTTTAC	CGAATATAACGCGACTAACTTACG
NY-ESO1	*Unmetilated*	CTACACACAAAAACCCTACTTCCA	TGGTATTGTGGTTATTTTTGGTTT
	Methylated	GCACAAAAACCCTACTTCCG	TTGCGGTTATTTTTCGGTTC

The copy number of methylated or unmethylated sequences for LINE-1, MAGE-A1 and NY-ESO-1 genes was established by extrapolation from the standard curves. The percentage (%) of methylation was defined as ratio between methylated molecules and the sum of methylated and unmethylated molecules and data were reported as % of LINE-1, MAGE-A1 and NY-ESO-1 methylation in treated and untreated cells.

Quantitative western blot analysis. Primary antibodies were diluted in milk 5% or BSA 5% in TBST and incubated overnight. Development was performed with the ECL normal western blot detection system by the chemiluminescence method. Images were acquired with the Alliance

Imaging System (Uvitec). Densitometric analysis was carried out by Quantity One software (Bio-Rad Laboratories Inc.). Normalized treated/control ratios were computed on the basis of background-adjusted density values and then visualized by a color code.

Data Analysis. The Transcriptomics Analysis Console (TAC) software (Applied Biosystems) was used to identify significantly modulated genes by treatment of two melanoma cell lines (VRG100 and CST30) with guadecitabine, givinostat, JQ1, OTX-015, GSK126 or abemaciclib, and of melanoma nodules removed from immunodeficient mice treated with Guadecitabine. Analysis settings were as follows: gene level fold change >|1.2|, gene-level p value: <0.05; gene-level FDR: <0.05. Ingenuity Pathway Analysis (IPA 8.5, www.ingenuity.com) was used to carry out Upstream Regulator (UR) analysis on: a) significantly modulated genes by different treatments; b) differentially expressed genes in pre-therapy and on-treatment lesions from responding vs non responding patients from the NIBIT-M4 trial (ref. 24 in the manuscript). Upstream regulator analysis allows to identify upstream transcriptional regulators that can explain the observed gene expression changes in the dataset. This computational tool returns results based on p-values and Z score statistics. P values indicate the likelihood of the association between a set of genes and related function, or the likelihood of the overlap between the genes in the dataset and those that are regulated by a predicted upstream regulator. The meaning of the Z score statistics is to infer the activation states ("increased" or "decreased") of the identified biological functions and of the predicted transcription factors. Only Z scores greater than 2 or smaller than -2 were considered significant. IPA was also used for canonical pathway analysis. Canonical pathway analysis is a computational tool allowing to determine if canonical pathways are activated or inhibited on the basis of gene expression in the dataset. Activation or inhibition states of canonical pathways are predicted based on the Z-score algorithm. The significance values (p-value of overlap)

are calculated by the right-tailed Fisher's Exact Test and indicate the probability of association of molecules in the dataset with the canonical pathway by random chance alone.
