# Supplementary material for: Landscape of immune-related signatures induced by targeting of different epigenetic regulators in melanoma: implications for immunotherapy
Source: J Exp Clin Cancer Res. 2022 Nov 17;41:325. doi: 10.1186/s13046-022-02529-5 (PMC9670381; doi:10.1186/s13046-022-02529-5)
Supplement: Supplementary file 2 — Additional file 2: Supplementary Figs. S1 to S21. Supplementary Fig. S1. Expression in ten melanoma cell lines of genes and gene families targeted by epigenetic drugs. Supplementary Fig. S2. Differentiation profile of melanoma cell lines. Supplementary Fig. S3. Susceptibility of ten melanoma cell lines to the anti-proliferative effects of epigenetic drugs. Supplementary Fig. S4. Methylation status of LINE-1, MAGE-A1 and NY-ESO-1 in 9 melanoma cell lines treated with guadecitabine. Supplementary Fig. S5. Volcano plot of differentially expressed genes in VRG100 and CST30 cell lines. Supplementary FigureS6. Whole genome gene modulation analysis by 4 epigenetic drugs in two melanoma cell lines. Supplementary Fig. S7. Edwards-VENN diagram analysis of significantly modulated genes by epigenetic drugs. Supplementary Fig. S8. Original and revised gene classification of the NanoString nCounter PanCancer Immune Profiling panel. Supplementary Fig. S9. Quantitative analysis of Nanostring data in ten melanoma cell lines treated with epigenetic drugs. Supplementary Fig. S10. Modulation of immune-related genes in melanoma cell lines by epigenetic drugs. Supplementary Fig. S11. Comparison of immune-related gene modulation by BET inhibitors JQ1 and OTX-015. Supplementary Fig. S12. Immune-related signature of epigenetic drugs in melanoma. Supplementary Fig. S13. Outline of the strategy for quantitative analysis and visualization of western blot data. Supplementary Fig. S14. Quantitative western blot analysis and visualization of the modulation of LMP7 by epigenetic drugs in 11 melanoma cell lines. Supplementary Fig. S15. Pipeline of data analysis based on Upstream Regulators (UR) identified by IPA. Supplementary Fig. S16. Classification of URs significantly modulated by at least two different drugs in melanoma cell line VRG100. Supplementary Fig. S17. Classification of URs significantly modulated by at least two different drugs in melanoma cell line CST30. Supplementary Fig. S18. Sum [file 13046_2022_2529_MOESM2_ESM.pdf]

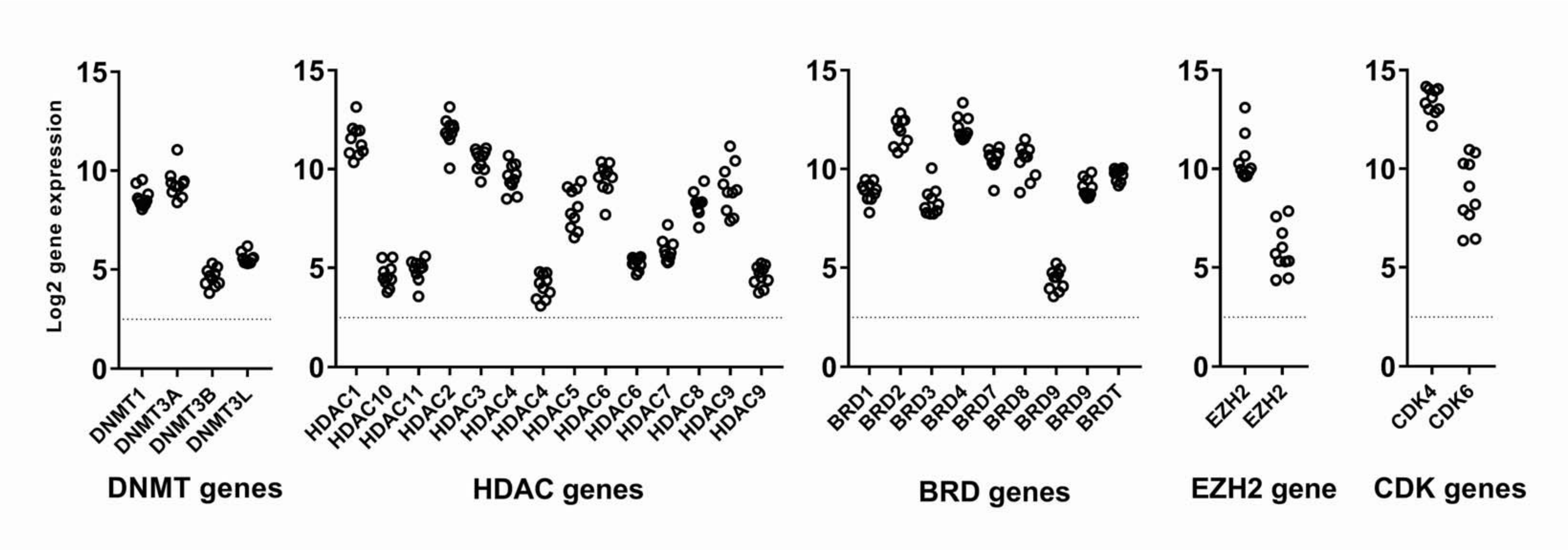

Supplementary Figure S1. Expression in ten melanoma cell lines of genes and gene families targeted by Decitabine/Guadecitabine (DNMT genes), Givinostat (HDAC genes), JQ1 or OTX-015 (BRD genes), GSK-126 (EZH2 gene, two different probes present for this gene in the Clariom S array), and Abemaciclib (CDK4/6 genes). Melanoma cells lines tested in this assay were: GML41, VRG100, BRM17, CST30, BNV13, CPN20, FRN39, GRD43, SLN91, MNT59.

Supplementary Figure S2

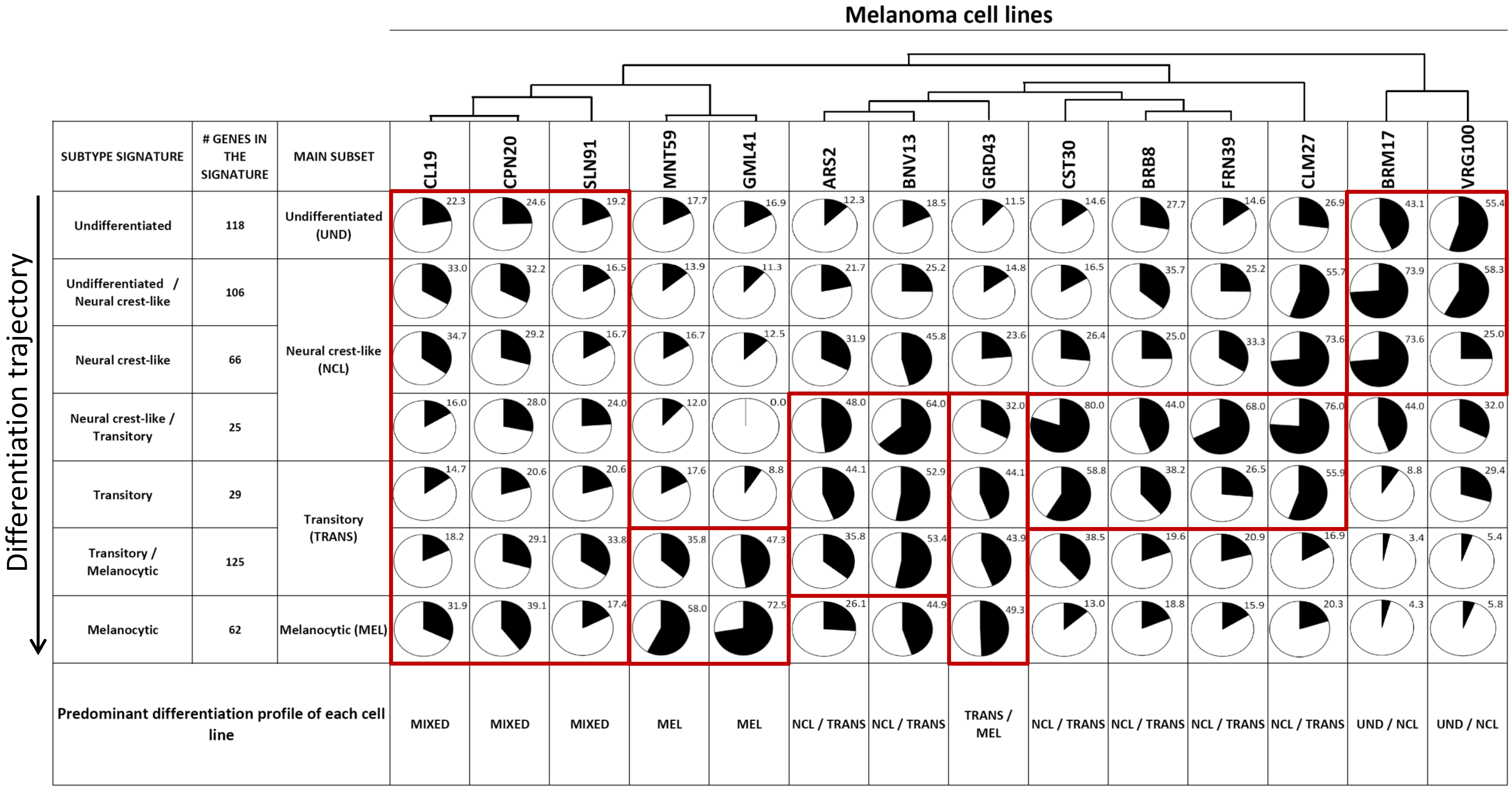

Supplementary Figure S2. Melanoma differentiation profile of cell lines according to expression of seven subtype signatures and four main melanoma subsets as defined by Tsoi et al. [33]. Expression of all genes belonging to each of seven subtype signatures was evaluated by Clariom S arrays. For each cell line the pie charts indicate the % of genes within each subtype signature that have median centered expression >0.5 (in Log2 space). Red rectangles highlight the predominant differentiation profile of each cell line. MEL: melanocytic; NCL/TRANS: neural crest-like / transitory; TRANS/MEL: transitory / melanocytic; UND/NCL: undifferentiated / neural-crest-like. Cell lines were clustered by Cluster 3.0 according to the expression of genes in each subtype signature.

Supplementary Figure S3

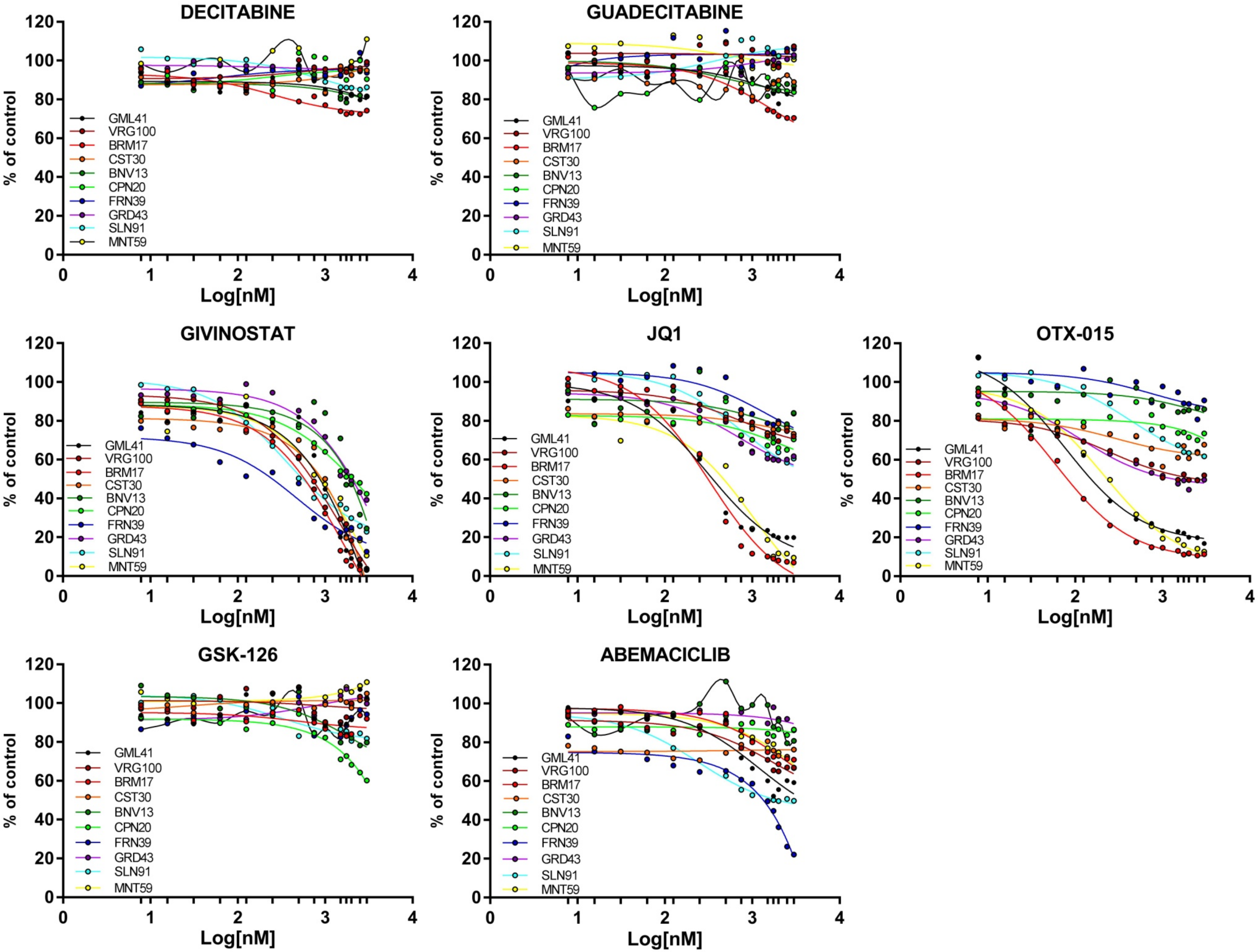

Supplementary Figure S3. Susceptibility of ten melanoma cell lines to the anti-proliferative effects of the indicated epigenetic drugs was evaluated at 96h by the MTT assays.

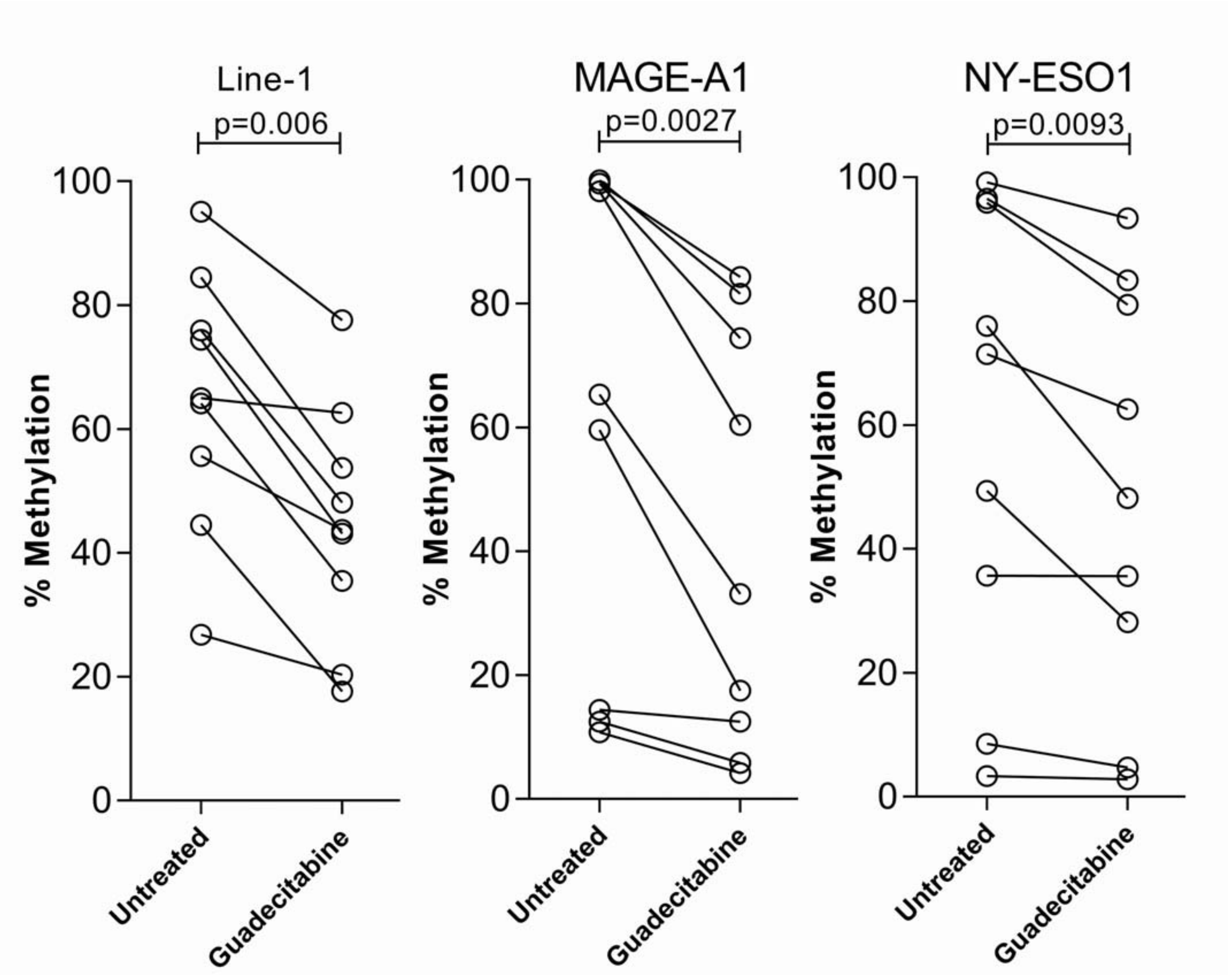

Figure S4. Methylation status of LINE-1, MAGE-A1 and NY-ESO-1 in 9 melanoma cell lines treated with guadecitabine. Genomic DNA was extracted from 9 melanoma cell lines (ARS2, BRB15, BRM17, CPN20, CST30, FRN39, GML41, SLN91, VRG100) either untreated or treated with guadecitabine as described in materials and methods and in Table S2. Real-time quantitative methylation-specific PCR analysis of LINE-1, MAGE-A1 and NY-ESO-1 promoters were performed on bisulfite modified genomic DNA using methylated- or unmethylated-specific primer pairs. Data are reported as % of LINE-1, MAGE-A1 and NY-ESO-1 methylation in guadecitabine-treated and untreated cells. Statistical analysis by paired T test.

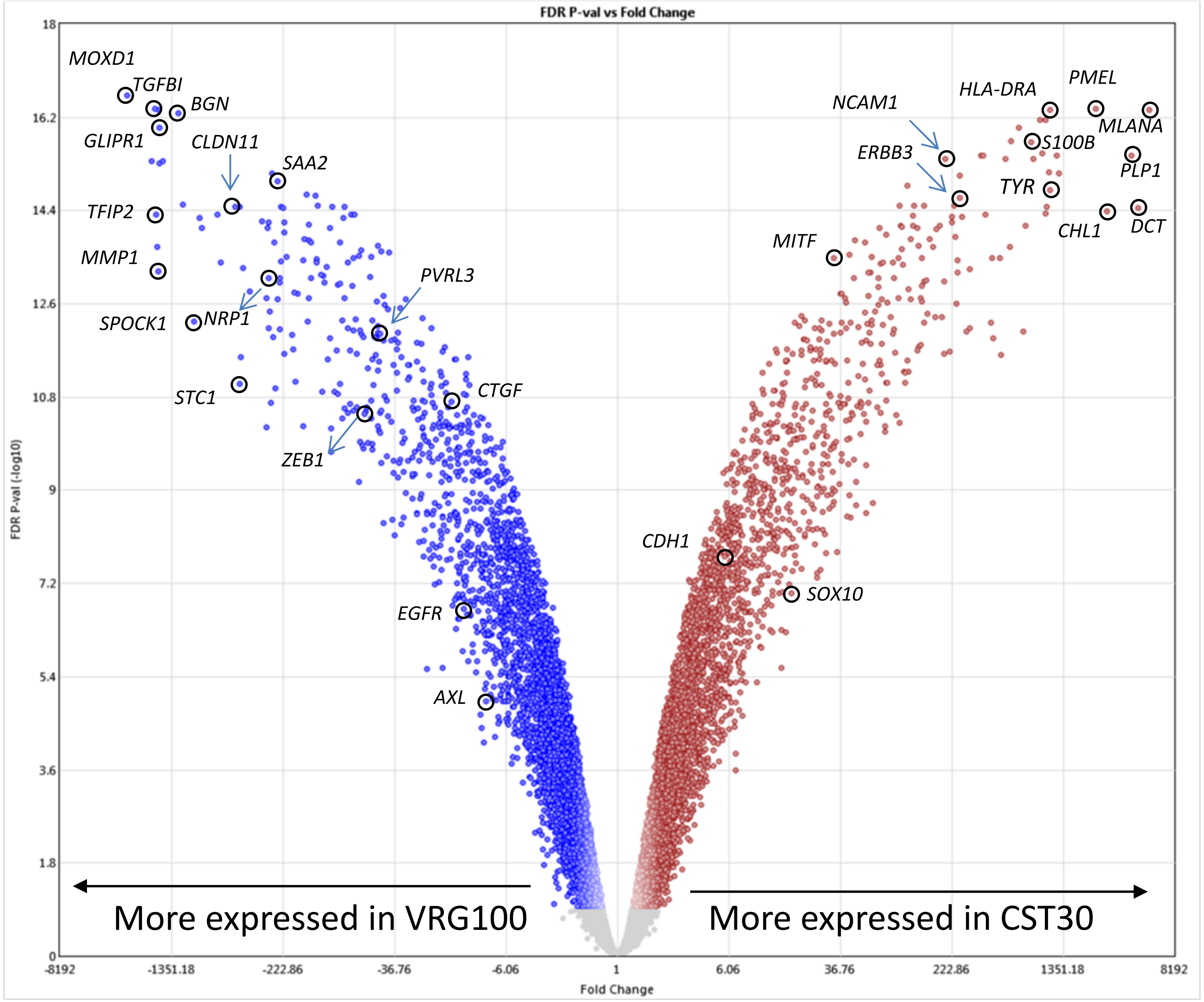

Supplementary Figure S5. Volcano plot of differentially expressed genes in VRG100 and CST30 cell lines. Genes identified by circles and gene symbols highlight the divergent phenotypic profile of the two cell lines, with CST30 showing higher expression of several genes associated with a more differentiated state (e.g. *MITF, SOX10, PMEL, MLANA, TYR, DCT, ERBB3*) and VRG100 showing higher expression of genes associated with a more undifferentiated/mesenchymal state (e.g. *AXL, EGFR, ZEB1, TGFBI, SPOCK1, PVRL3, CTGF).*

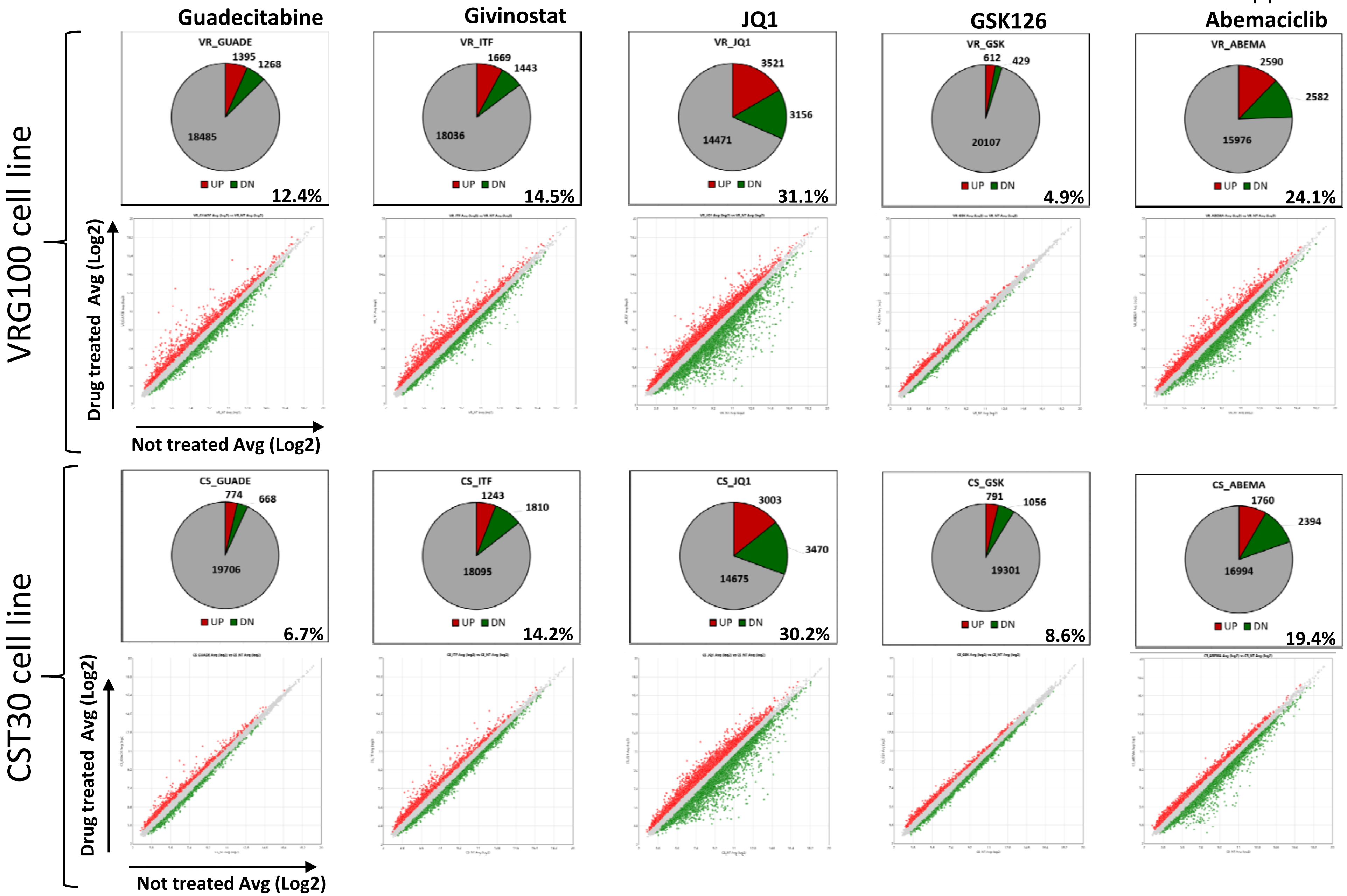

Supplementary Figure S6. Whole genome gene modulation analysis by 4 epigenetic drugs (guadecitabine, givinostat, JQ1, GSK126) and by a control drug (abemaciclib) in two melanoma cell lines (top graphs: VRG100; bottom graphs: CST30). For each cell line and drug, quantitative data of gene modulation are shown as pie charts indicating the number of up-regulated (red), down-regulated (green) or not modulated (grey) genes and the % of genes passing the filter (FC |>1.2|, $p<0.05$). Scatter plots show extent of gene modulation by each drug (red: upregulated genes, green: downmodulated genes) in the two cell lines.

Supplementary Figure S7

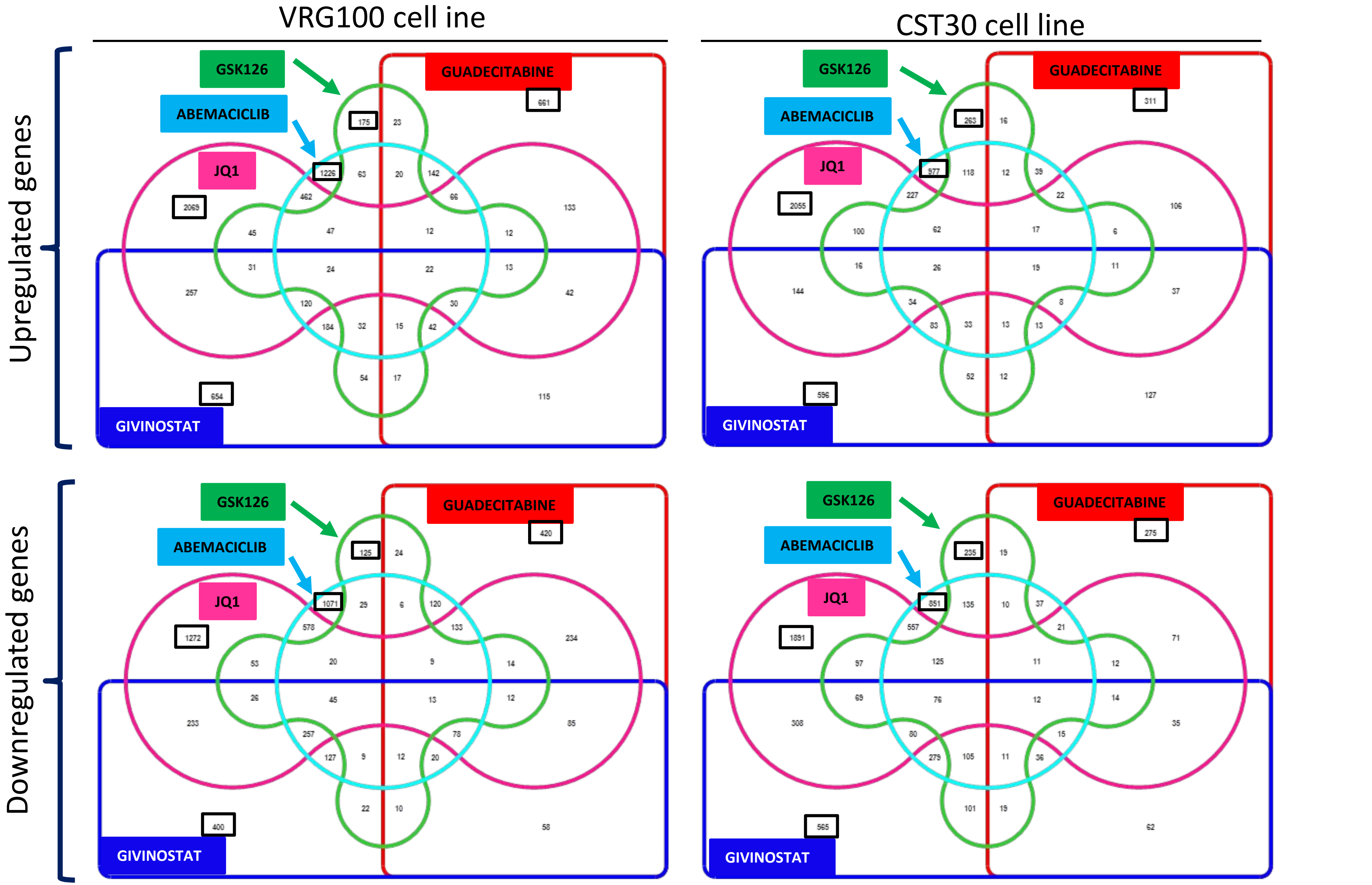

Supplementary Figure S7. Edwards-VENN diagram analysis of significantly modulated genes (upper panels, upregulated genes; lower panels, downregulated genes) in VRG100 (left hand panels) and CST30 (right hand panels) cell lines treated with guadecitabine (red rectangle), givinostat (blue rectangle), JQ1 (fuchsia peanut shape), GSK126 (green cogwheel) or abemaciclib (light blue circle). Numbers highlighted by a black frame represent genes modulated only by each of the drugs. All other numbers at the intersection of different colour-coded shapes represent genes co-modulated by more than one drug.

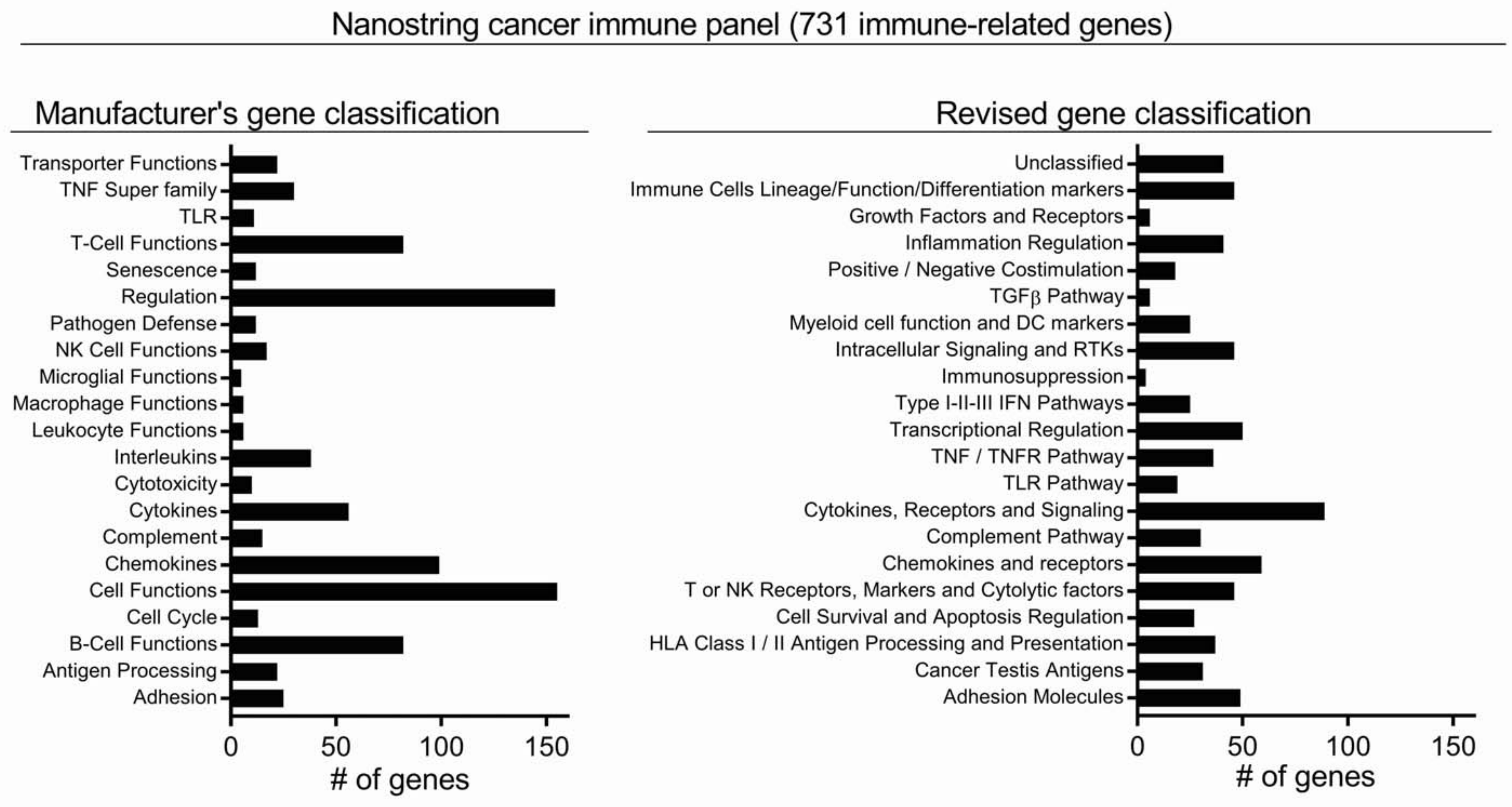

Supplementary Figure S8. Original (manufacturer's classification) and revised gene classification of the NanoString nCounter PanCancer Immune Profiling panel. All genes in the Nanostring panel were re-classified for function by accessing the human gene database Genecards (at http://Genecards.org) and through literature search.

Supplementary Figure S9

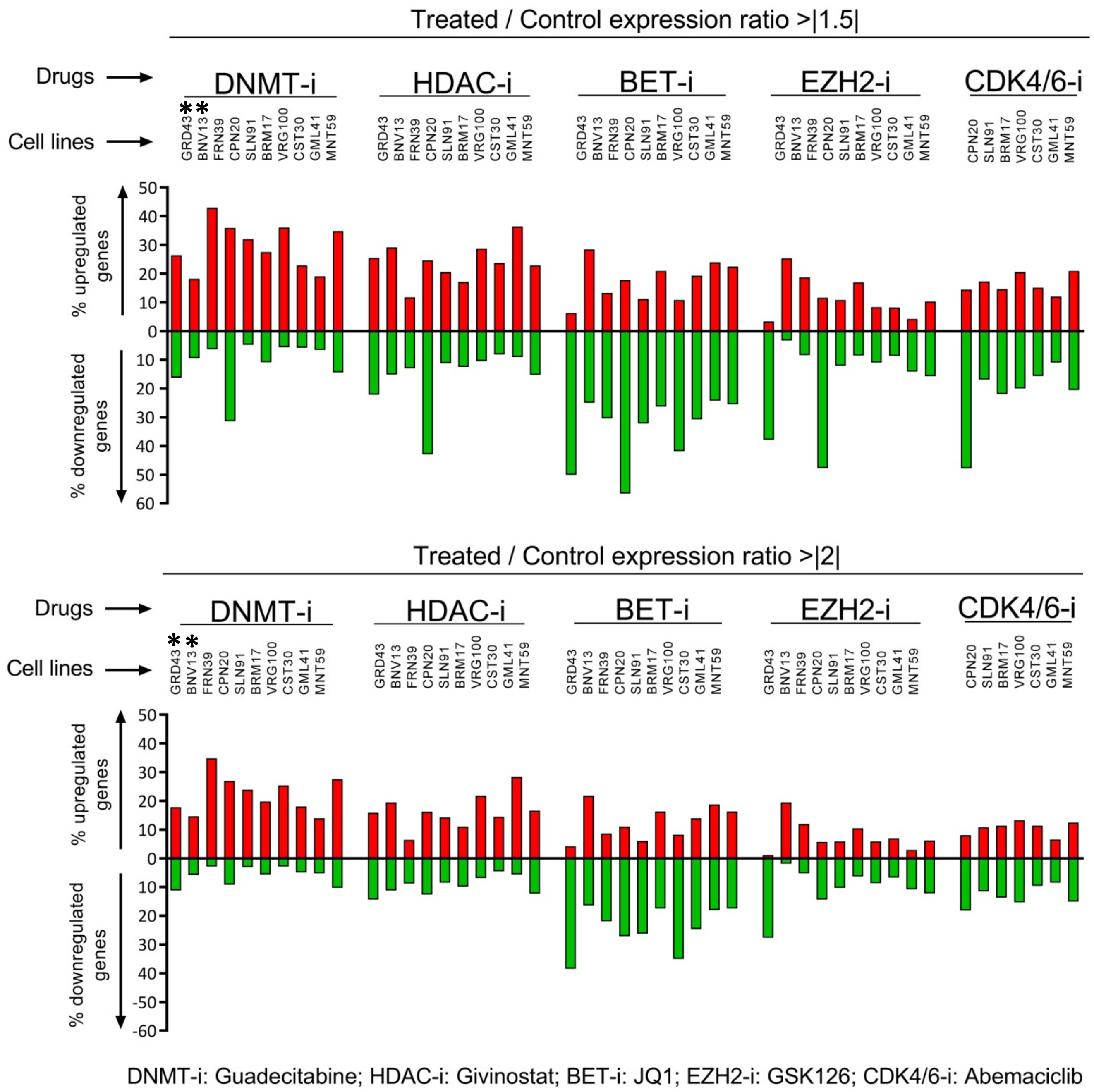

Supplementary Figure S9. **Quantitative analysis of Nanostring data in ten melanoma cell lines treated with the indicated drugs**. DNMT-i: decitabine / guadecitabine; HDAC-i:Givinostat; BET-i: JQ1; EZH2-i: GSK-126; CDK4/6 i: Abemaciclib. Upper graphs: % of genes in the Nanostring panel upregulated (red histograms) or downmodulated (green histograms) with a treated/control expression ratio >|1.5|. Lower graphs: % of genes upregulated (red histograms) or downmodulated (green histograms) with a treated/control expression ratio >|2.0|. *: these two cell lines were treated with decitabine, the active metabolite of guadecitabine.

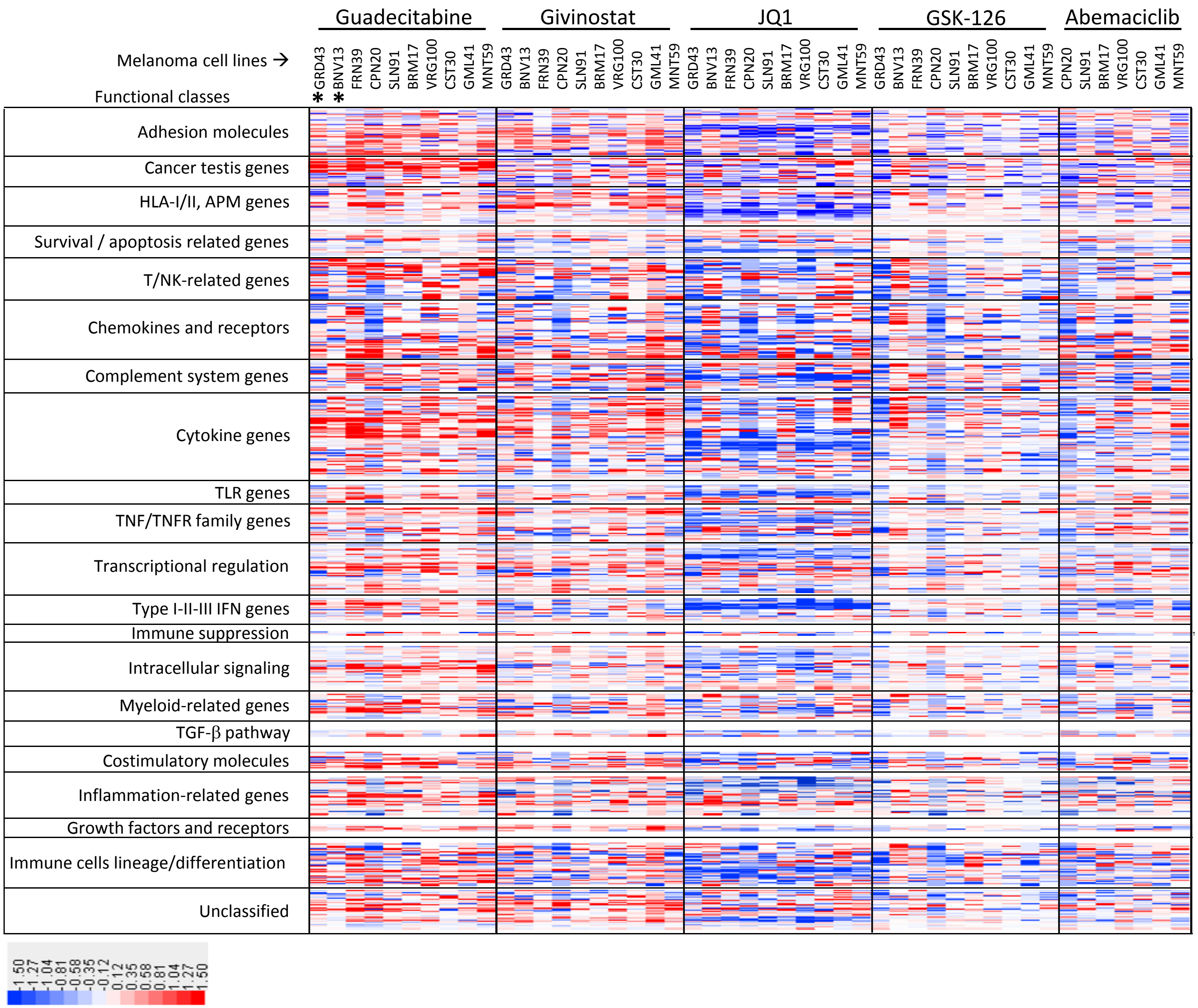

Supplementary Figure S10. Modulation of immune-related genes in melanoma cell lines by epigenetic drugs. Modulation of 731 genes in ten melanoma cell lines was assessed by the Nanostring Cancer Immune panel upon treatment with 4 epigenetic drugs and with the control drug Abemaciclib. Genes were clustered according to each of 21 functional classes. *: These two cell lines were treated with decitabine, the active metabolite of guadecitabine.

Supplementary Figure S11

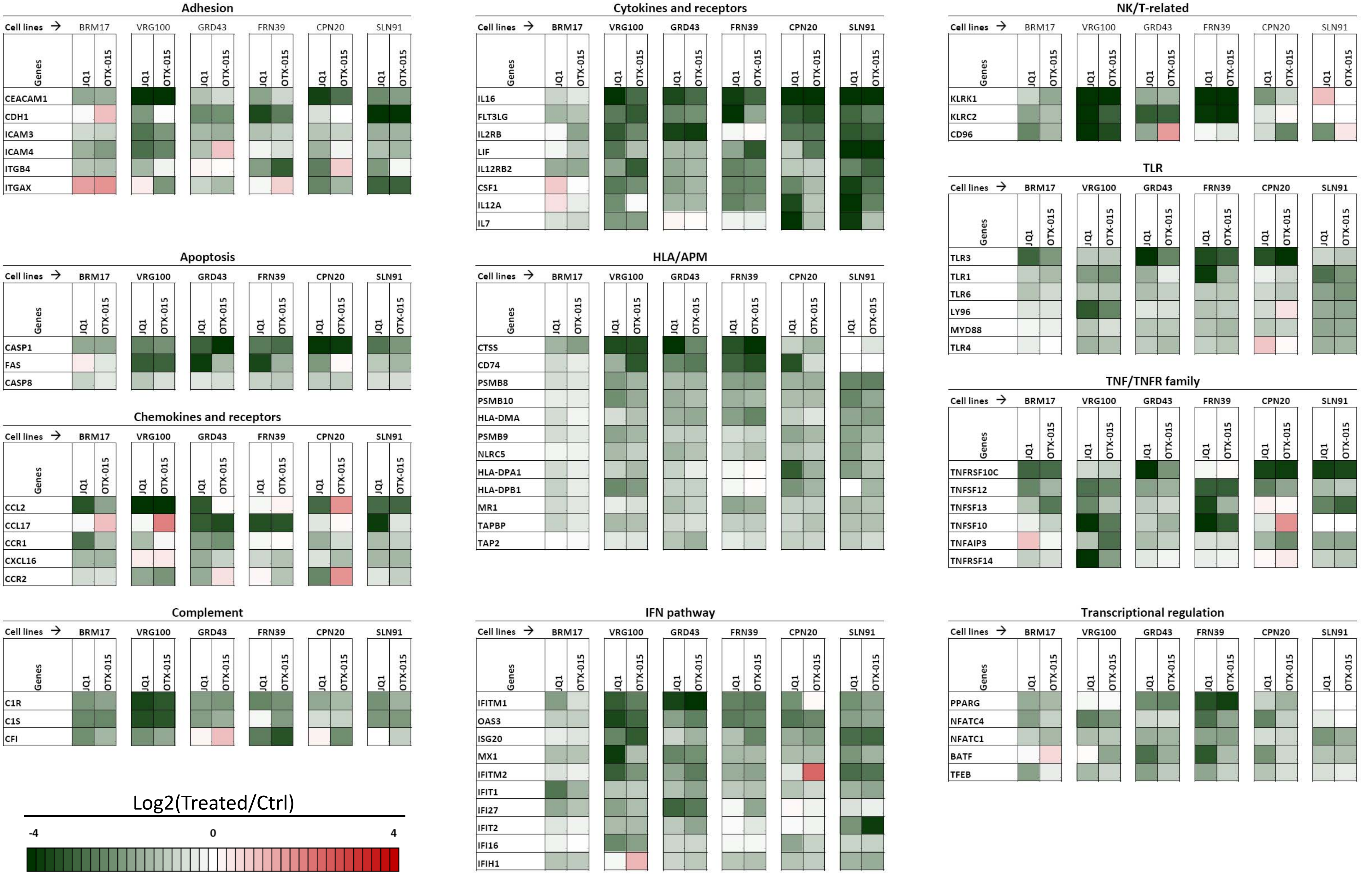

Supplementary Figure S11. Comparison of immune-related gene modulation by BET inhibitors JQ1 and OTX-015. Modulation of selected genes within 11 functional classes by JQ1 and OTX-015 was assessed by the Nanostring Cancer Immune Panel.

Supplementary Figure S12

GENE CLASS	GUADECITABINE	GIVINOSTAT	JQ1	GSK-126
Cancer testis	SSX1, MAGEB2, SSX4, CTCFL, CT45A1, CTAG1B, MAGEC2, DDX43, MAGEC1, MAGEA1, PASD1	TMEFF2, MAGEC2, CTCFL, MAGEC1, TTK, PBK	CT45A1, BAGE, PNMA1, PBK, CTCFL	SSX1, CT45A1
Class I/II Antigen Processing and Presentation	CD74, HLA-DMB, HLA-DPA1, HLA-DRB4	CD74, HLA-DRB3, HLA-DPA1, HLA-DMB, HLA-DPB1, HLA-DRB4, CD1D, HLA-DMA, HLA-DRA, MICB, HLA-B, HLA-DOB, PSMB10	CD1C, CD1D, TAPBP, HLA-DRA, NLRC5, HLA-DMA, PSMB9, HLA-DPA1, PSMB10, CD74, PSMB8, CTSS, TAP2, HLA-B, MR1, HLA-DRB4, TAP1	
Type I-II-III IFN system	IFI27, IFITM1, ISG20, IFNL2, IFITM2, IFNA7	IFITM1, IFNA7	IFITM1, MX1, OAS3, IFITM2, IFIT1, IFI27, ISG20, ISG15, IFIH1, IFI16, IFIT2	IFNG
Chemokines and receptors	CCL20, CXCL11, XCL2, CCR1, CXCL5, CCL2, CXCL2, CXCR6, CXCR4, CXCL14	CXCR4, CCL7, CCR6, CCL11, PPBP, CXCR3	CXCL3, CXCL2, CX3CR1, CCR2, CXCR3, CXCL13, XCR1, CXCL9, CXCL16, CCR1, CCL2, CCL17	PPBP, CCL11, CCR6
Cytokines,receptors and signaling	IL11, IL2RG, IL24, IL13RA2, IL1B, IL2RB, IL17B, Il18, Il10, IL17RB, IL1A, IL32, CSF2, IL22RA2, IL4R, IL23A, STAT4, IL8, LIF, IL15RA, IL6, IL22RA1, IRAK2, IL1RL1, IL18R1, IL1R2	IL1B, SPP1, IL1A, Il8, IL21R, IL7R, IL10RA, IL15, IL15RA, IL3RAIL12RB1, JAK3, IL16, IL1RL1, SIGIRR	IL12RA, IL8, IL17A, IL16, FLT3LG, IL12A, LIF, IL7, CSF1, IL12RB2, IL6, SIGIRR, JAK3, IL21R, IL1R1, IL24, IRAK1, OSM, IL4R, STAT6, IL6R, IL1RAP, IL13RA2, IL34, IL1RAPL2	IL2RB, SIGIRR, IL3RA, IL1RL1, IL32
TNF Super family	TNF, TNFRSF10C, TNFSF10, TNFSF11, TNFRSF1B, CD70, TNFRSF18, TNFSF15, TNFSF4, TNFSF13	CD70, TNFRSF11A, TNFSF10,TNFRSF1B, TNFRSF18, TNFSF15, TNFSF8	TNFRSF17, TNFRSF12A, TNFRSF8, TNFSF12, TNFRSF10C, TNFSF13, TNFSF10, TNFRSF14, TNFSF15,TNFSF13B, TNFAIP3, LTBR, TNFRSF11A, TRAF3, TNFSF11, TNFRSF13C	
POS-NEG costimulation	CTLA4, LAG3, ICOSLG, TIGIT, HAVCR2	CD274, CD40, HAVCR2, CD40LG	HAVCR2, PDCD1LG2, CD200, CD40, CD80, CD274, TIGIT	LAG3, CD40LG
Adhesion	PECAM1, ITGB4, SELPLG, ITGB2, ITGB3, ICAM2, ITGA2B, ITGAX,THBS1, EPCAM	ITGAX, SELL, ITGA2B, ALCAM, NCAM1, FN1, ITGB3, THBS1, TH1, ITGB4	CLEC5A, ITGB3, ALCAM, ITGA6, CD97, ITGAX, THY1, EPCAM, ICAM3, CDH1, CEACAM1	ITGA2B, SELPLG, AMICA1
Complement system	CFI, CFB, C4B, C8G, C6, MBL2, SERPING1, MASP1	CFD, CFI, C3, SERPING1, C1R	C8B, C1R, C1S, SERPING1, CFD, C2, CFI, CD55, CFB	MASP1, C6
TLR system	TLR4, TLR9, TLR2	TLR5	TLR10, TLR8, TLR9, TLR5, TLR3, TLR1, TLR6, LY96, MYD88, TLR4, TICAM2	
Intracellular signaling and RTKs	PIK3CG, INPP5D, ZAP70, AXL, SERPINB2, SOCS1, SH2D1B, TXK, LCK	SERPINB2, HCK, TXK, SH2D1B, INPP5D, LCK	SOCS1, HCK, MERTK, INPP5D, BTK, MAPK3, MAP3K5, SH2D1B, ITK, MAPK11, PIK3CD, AXL, LCK, MAP3K1	HCK, SH2D1B, SERPINB2
Transcriptional regulation	RORC, POU2F2, FOXJ1, EGR2, IRF7, PAX5, MNX1	EGR1, EGR2, MAF, IRF5, CREB5, TCF7, FOXP3, PAX5	MAF, EGR2, AIRE, IRF7, IRF4, PPARG, NFATC4, BATF, NFATC1, EOMES, TFEB, NFATC2, CEBPB, TP53, CREBBP, ATF1, PAX5, FOXP3	FOXJ1, TBX21, PAX5, AIRE
Inflammation regulation	SAA1, F2RL1, S100A7, NOS2A, PTGS2, CAMP, SPINK5, ANXA1, TXNIP, NOD2, ADORA2A, NLRP3, MEFV	S100A7, NOD2, F2RL1, APOE, PTGS2, TXNIP, NOS2A, PLA2G1B, MEFV, PYCARD	TXNIP, S100B, S100A7, SPINK5, IKBKE, PYCARD, CAMP, APOE, LRP1, ANP32B, ANXA1	MEFV, PLA2G1B, ADORA2A
Cell survival and apoptosis regulation	ATM, BCL6, CLU	ATM, CYFIP2, BCL6, CARD11, FAS, BIRC5, CLU	ATM, ATG12, CDKN1A, BID, BCL2L1, CASP8, BIRC5, FAS, CASP1	
T or NK receptors, markers and cytolytic factors	DPP4, CTSW, CD96, PRF1, SPN, CD4, KLRC2, GZMM, CD244, CD38, LCP1	KLRK1, KLRC2, GZMB, CD96, LCP1, KIR_Inhibiting_Subgroup_1, CD244, KLRD1	ENTPD1, CD8B, CD244, CD4, PTGDR2, DPP4, CD96, KLRK1, KLRC2	KIR3DL2, KIR_Inhibiting_Subgroup_1, GZMH, CD8B
Immune cells lineage/function/differ. markers	FCER1G, LY9, SLAMF1, SLAMF7, BST2, CD22, CD34, CD5, SH2D1A, SLAMF6, FCGR3A, CD79B, PTPRC, MS4A2	CD24, RAG1, CD36, PTPRC, PRG2, CD22, LTF, FCERIG, MS4A2, FCER2	BST2, SLAMF7, FCER1G, CD36, LTF, ADA, FCGR2A, BST1, RAG1, CD22, LILRB2, MME, FCER2, CD83, PLA2G6, MS4A2, FCER1A	CD5, FCER2, FCER1G, IGLL1, FCGR3A, FCER1A
Myeloid cell function and DC markers	CD14, TREM2, MST1R, THBD, NRP1, SLC11A1, CD207, CHIT1	CD14, TREM2, NRP1, CHIT1	CHIT1, NRP1, TREM2	MST1R, NCF4
NOT classified	LCN2, LAMP3, LRRN3, DMBT1, TPSAB1, PMCH, PLAU, PLAUR, SMPD3, HSD11B1, FPR2,FUT7, MUC1	NEFL, ABCB1, LAMP3, FEZ1, DMBT1, PLAUR, F12, MUC1	FPR2, NEFL, ABCB1, LAMP2, MUC1, F12, CTSH, CTSG, CTSL, SYT17, TPSAB1, PLAUR, DMBT1	F13A1

Supplementary Figure S12. **Immune-related signature of epigenetic drugs in melanoma**. The table shows the genes observed upregulated (red) or downmodulated (green) with the same direction of change in at least 6/10 cell lines and showing a Treated/Ctrl ratio >|1.5|.

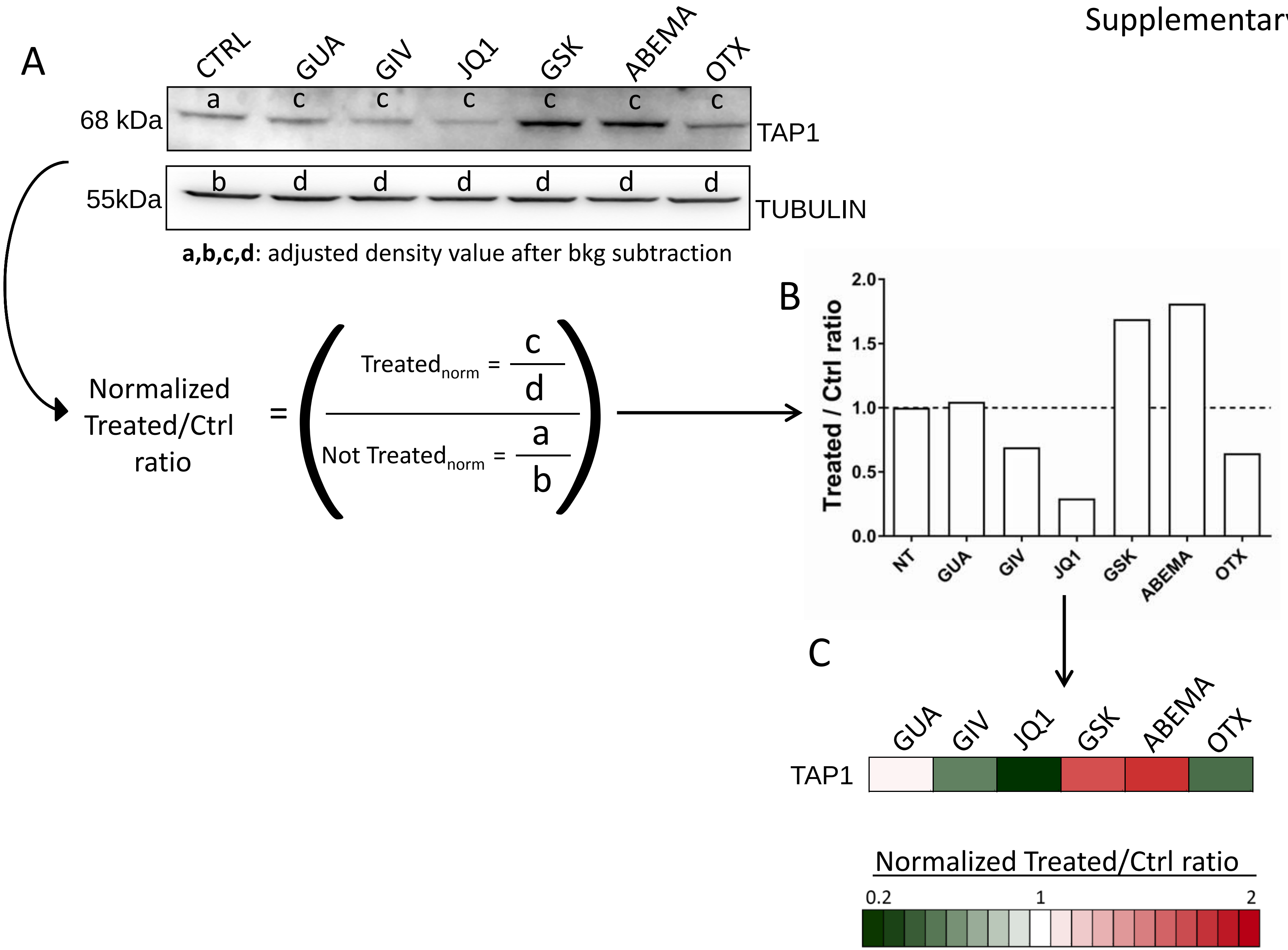

Supplementary Figure S13. **Outline of the strategy for quantitative analysis and visualization of western blot data. A.** Normalized treated/control ratios were computed on the basis of background-adjusted density values. **B,C**. Treated/control ratio values were then converted to a color-coded strip allowing direct visualization of the effect of each drug on markers of interest. CTRL. Untreated cells; GUA: guadecitabine; GIV: givinostat; GSK: GSK126; ABEMA: abemaciclib; OTX:OTX-015.

Supplementary Figure S14

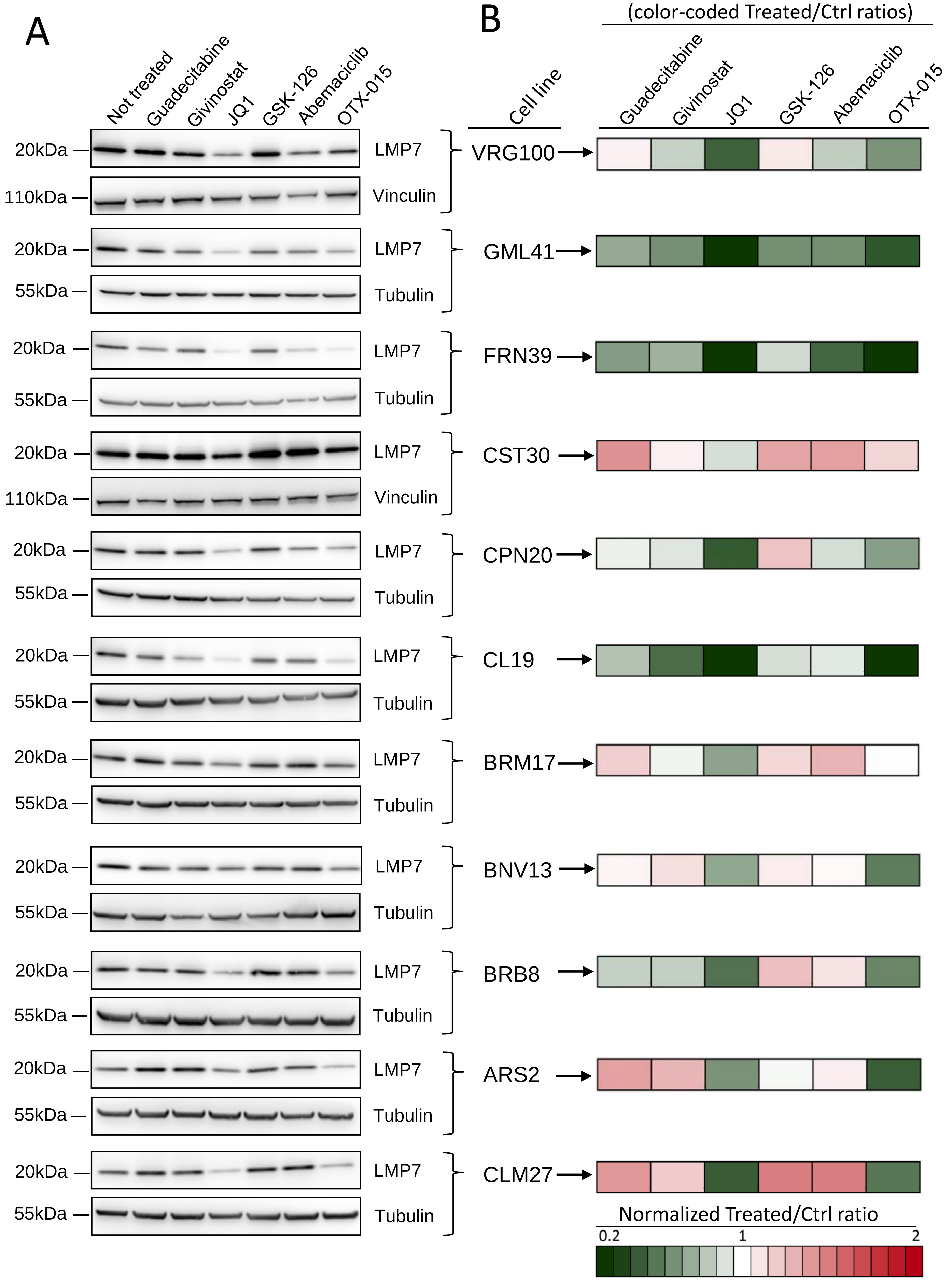

Supplementary Figure S14. Quantitative western blot analysis and visualization of the modulation of LMP7 by epigenetic drugs in 11 melanoma cell lines. **A**. Original western blot images. **B**. Color-coded normalized treated/control ratios as defined in Suppl. Fig. S13.

Supplementary Figure S15.

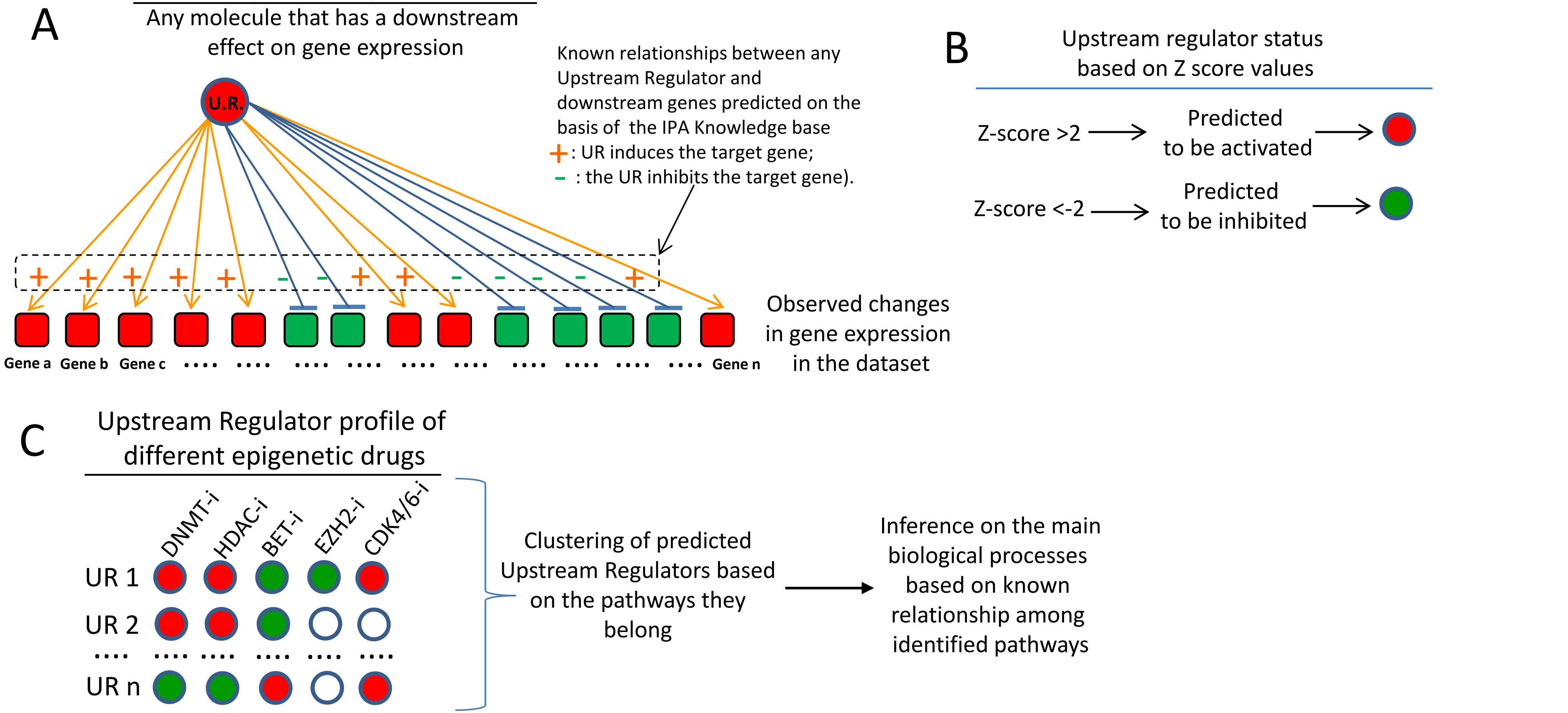

Supplementary Figure S15. **Pipeline of data analysis based on Upstream Regulators (UR) identified by IPA**. **A**. In this schematic, squares represent genes, while circles represent URs. Circle color denotes predicted UR status (red: activation; green: inhibition). Square color denotes observed gene expression change (red: upregulation; green: downmodulation). An UR is any molecule that can have a downstream effect on gene expression. The IPA knowledge base, built into the application, identifies the relationships between any set of genes being observed as significantly modulated in the dataset and the UR that controls them (relationship measured through a P value of overlap between a set of genes and any given upstream regulator). **B**. Depending on the type of relationship between the set of genes and an UR, and on the observed changes in gene expression, IPA computes a Z score statistics whose meaning is to infer the activation status ("activated" or "inhibited") of the UR. Only Z scores greater than 2 or less than -2 were considered significant. **C**. For each drug, the overall UR profile can be identified in terms of identity of the molecules (UR1, UR2…URn) and of their predicted activation status. Different URs can then be clustered together based on the common biological pathways they belong. Finally, an inference can be made on the biological processes being modulated by each drug based on known relationships between the identified pathways.

Supplementary Figure S16

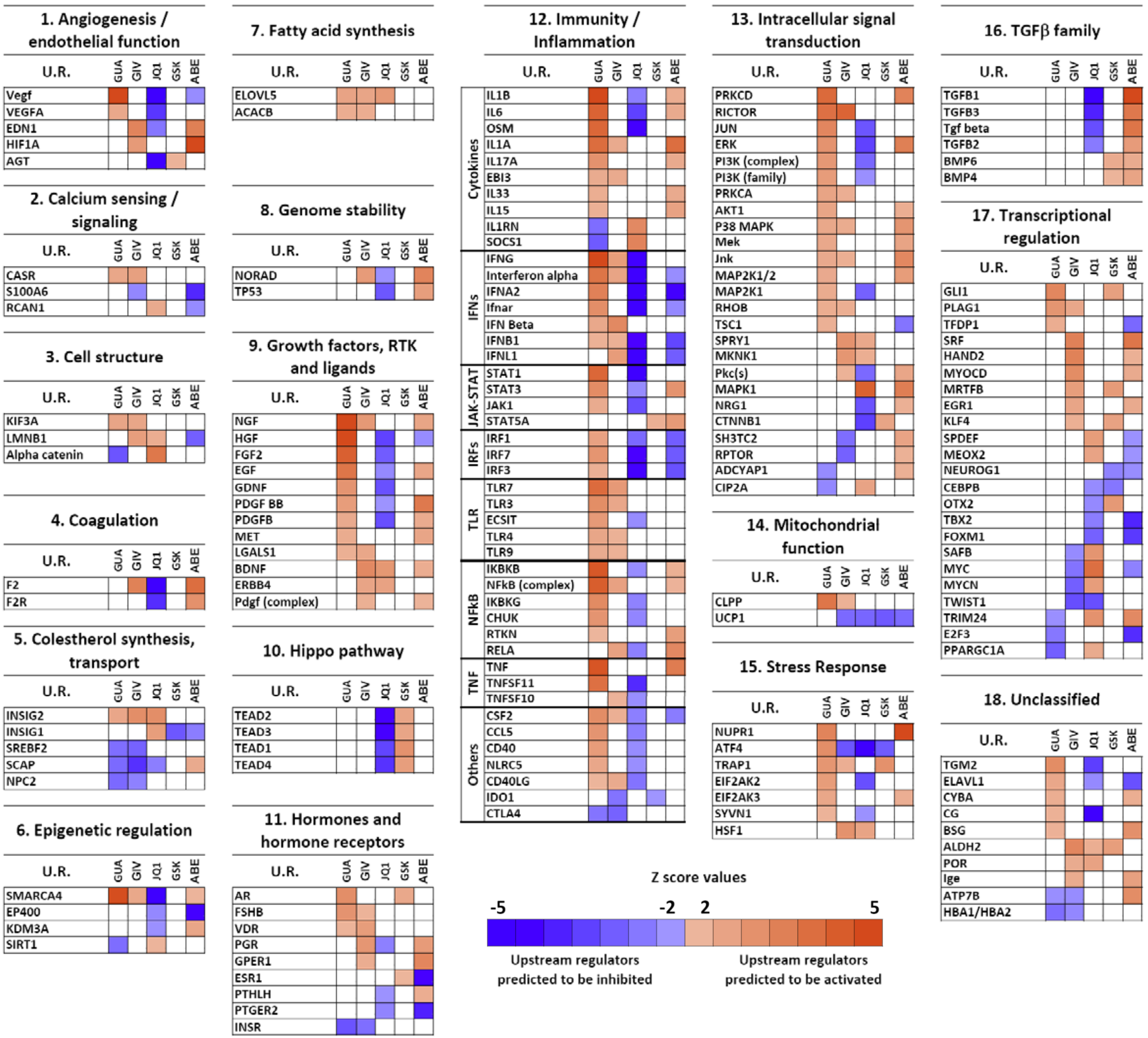

Supplementary Figure S16. Classification of URs significantly modulated by at least two different drugs in melanoma cell line VRG100. URs significantly modulated by at least two different drugs were grouped into 18 functional classes. Each UR was selected based on a significant Z score (>|2| and a significant p value for association with specific sets of modulated genes by each drug. Z score values of each UR are shown by a color code indicating prediction of UR inhibition (blue) or prediction of UR activation (red). GUA: guadecitabine, GIV: givinostat, GSK: GSK-126; ABE: abemaciclib.

Supplementary Figure S17

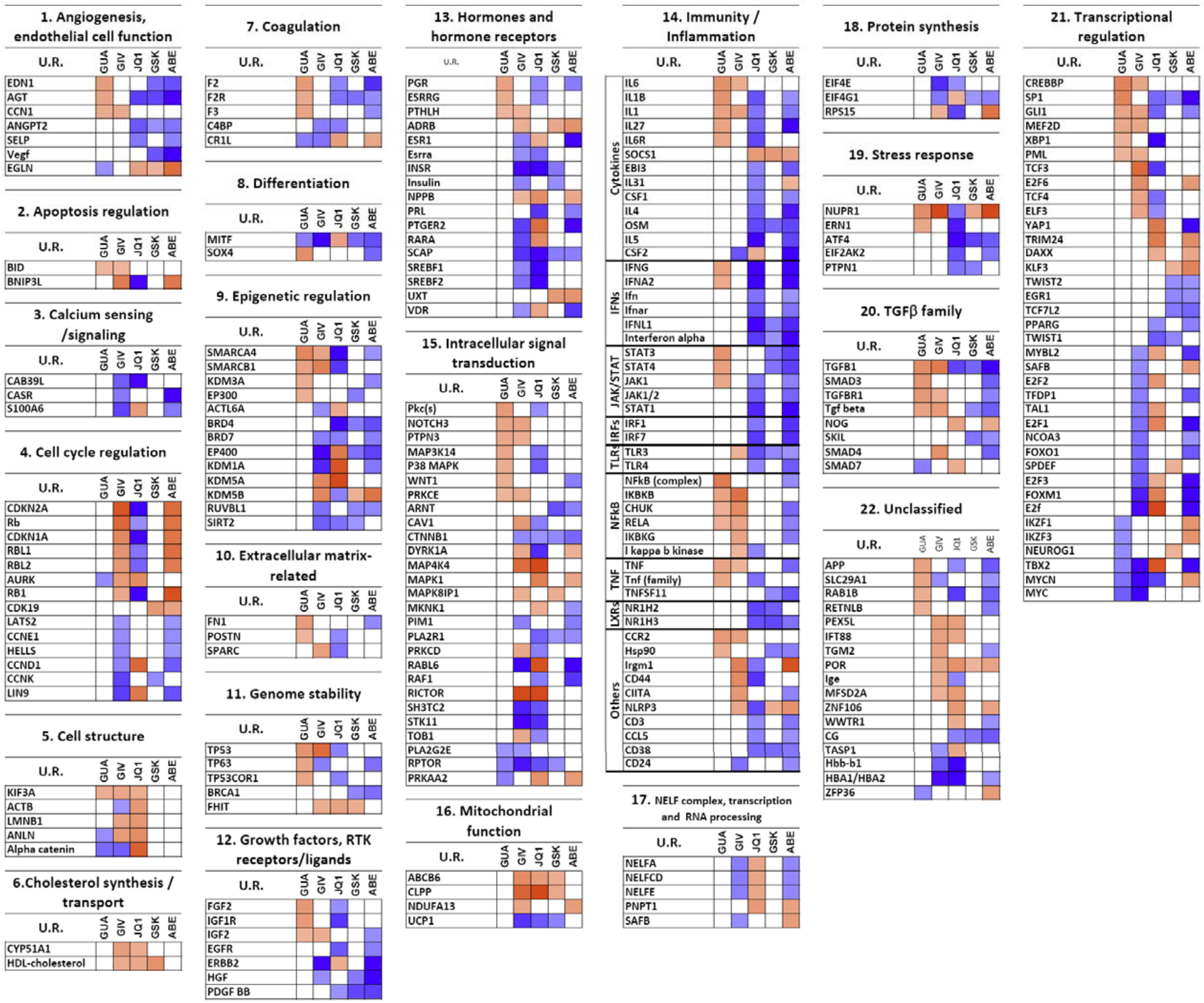

Supplementary Figure S17. Classification of URs significantly modulated by at least two different drugs in melanoma cell line CST30. URs significantly modulated by at least two different drugs were grouped into 18 functional classes. Each UR was selected based on a significant Z score (>|2| and a significant p value for association with specific sets of modulated genes by each drug. Z score values are shown by a color code indicating prediction of UR inhibition (blue) or prediction of UR activation (red). GUA: guadecitabine, GIV: givinostat, GSK: GSK-126; ABE: abemaciclib.

Supplementary Figure S18

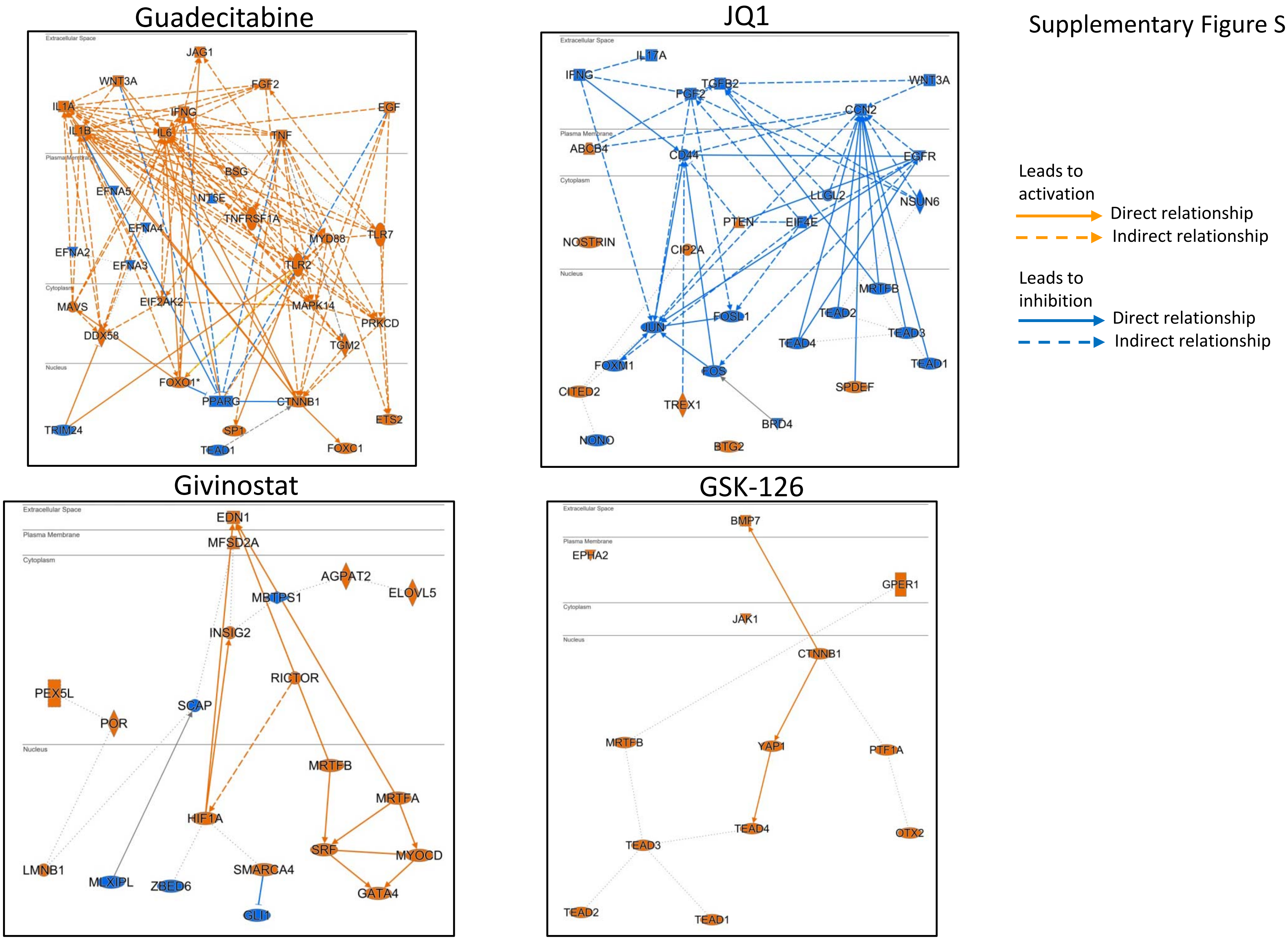

Supplementary Figure S18. Summary of the major functional networks linking relevant upstream regulators emerging from IPA Core Analysis of genes modulated by four epigenetic drugs in the melanoma cell line VRG100. The graphical abstract algorithm selects and connects the most significant Upstream Regulators (UR) emerging from the IPA Core analysis. URs are shown according to subcellular localization. URs have a color code reflecting the activation Z-score value: URs predicted to be activated have a positive z-score ($Z>2$, $p<0.05$) and are colored orange; URs predicted to be inhibited have a negative z-score ($Z<-2$, $p<0.05$) and are colored blue. Relationships among URs are shown by color coded arrows as indicated in the legend.

A

		Shared upstream regulators activated by Guadecitabine in-vivo and in-vitro	
		In vivo	In vitro
Pathway	Upstream Regulator	Z score values, tumor nodules from treated vs control mice, cell line 195	Average Z score values, treated vs untreated melanoma cell lines
	IFNG	7.05	4.20
	IFNA2	6.83	3.24
	IFNL1	6.28	3.14
	IRF1	5.26	2.78
	Ifnar	5.06	3.05
	STAT1	4.97	3.68
	EIF2AK2	4.88	2.85
	SMARCA4	4.70	2.75
	IRF3	4.40	2.85
	TGM2	4.08	2.89
	DOCK8	3.30	3.03
	TNF	3.26	4.60
	TICAM1	3.19	3.56
	IL1B	3.15	4.06
	SASH1	3.13	3.07
	SAMSN1	2.84	3.15
	RELA	2.56	3.37
	IKBKB	2.53	3.30
	DDX58	2.53	2.51
	TLR7	2.50	3.71
	TLR3	2.30	3.84
	CD40LG	2.23	3.00
	ARHGAP21	2.11	2.59

Code	Pathway
	Type-I/III IFN
	NF-kB
	TLR

Canonical pathway “Interferon signaling”

B

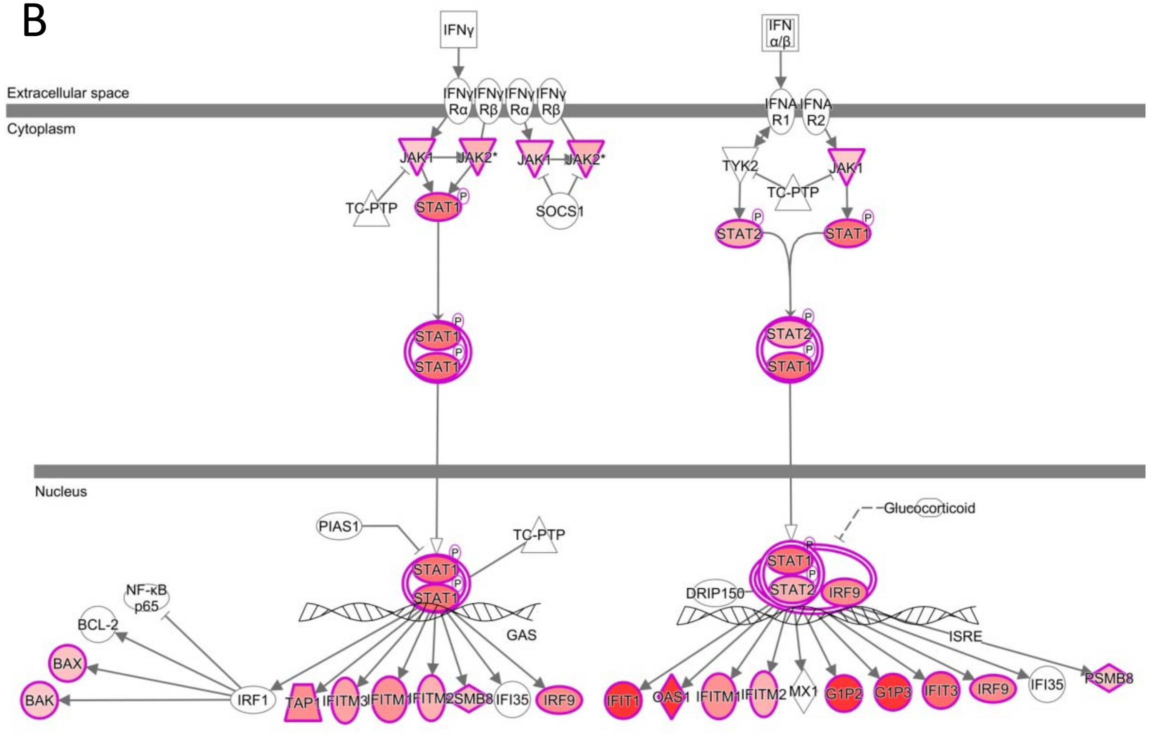

Supplementary Figure S19. Comparison of URs activated by guadecitabine in-vitro and in-vivo. A. Table of top URs activated by guadecitabine in tumor nodules from mice bearing a human melanoma xenograft (cell line 195) and treated with this drug. Z score values computed from gene expression data of treated mice vs control mice (first column) are compared, for each UR, to the average Z score value observed in vitro in melanoma cell lines treated with guadecitabine. **B**. Canonical pathway analysis of IFN-γ and IFN-α/β pathways modulated by guadecitabine in vivo in tumor nodules from treated vs control mice. Genes highlighted in red were observed as significantly upregulated by Guadecitabine.

Supplementary Figure S20

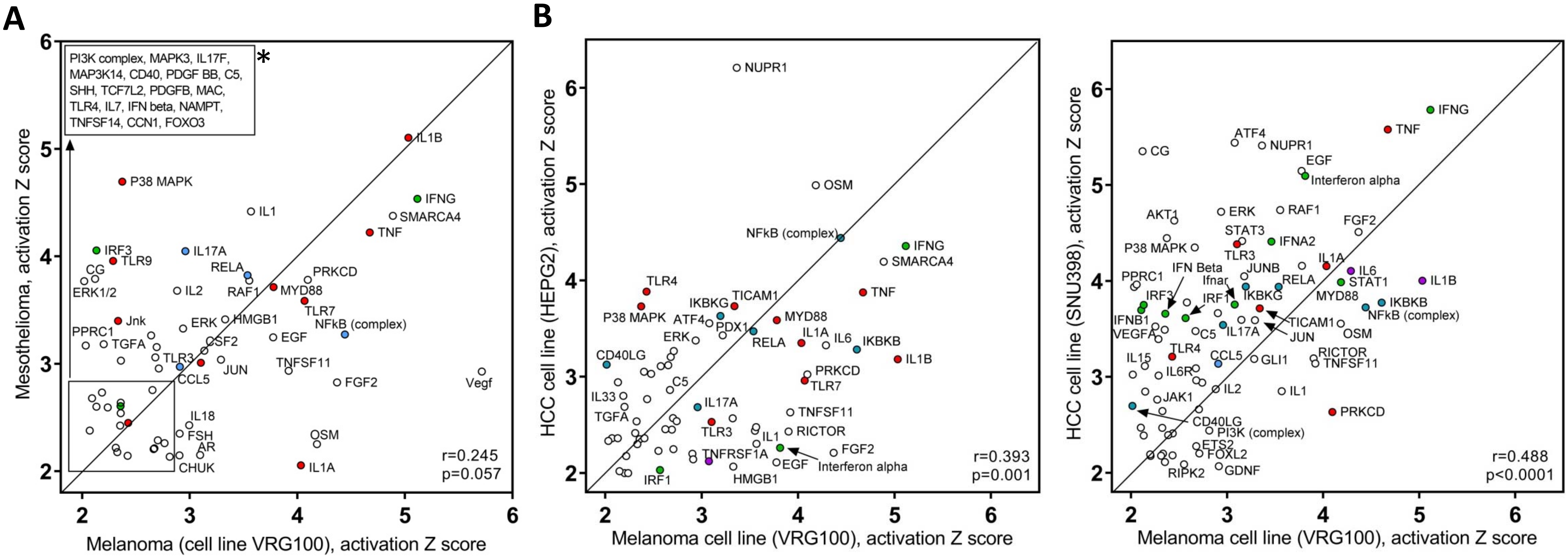

Supplementary Figure S20. Comparison of Upstream Regulators activated by guadecitabine in melanoma cell line VRG100 vs a mesothelioma cell line and in melanoma cell line VRG100 vs hepatocarcinoma cell lines HEPG2 and SNU398. A, B. Scatter plot of URs activated by guadecitabine in melanoma vs mesothelioma (A) and in melanoma vs hepatocarcinoma cell lines (B, according to gene expression data retrieved from ref. 36). Guadecitabine UR signature molecules shown in this figure are highlighted with the same color code used in Fig. 5 to mark the biological function/pathway. *: identity of URs shown in the square in panel A.

Supplementary Figure S21

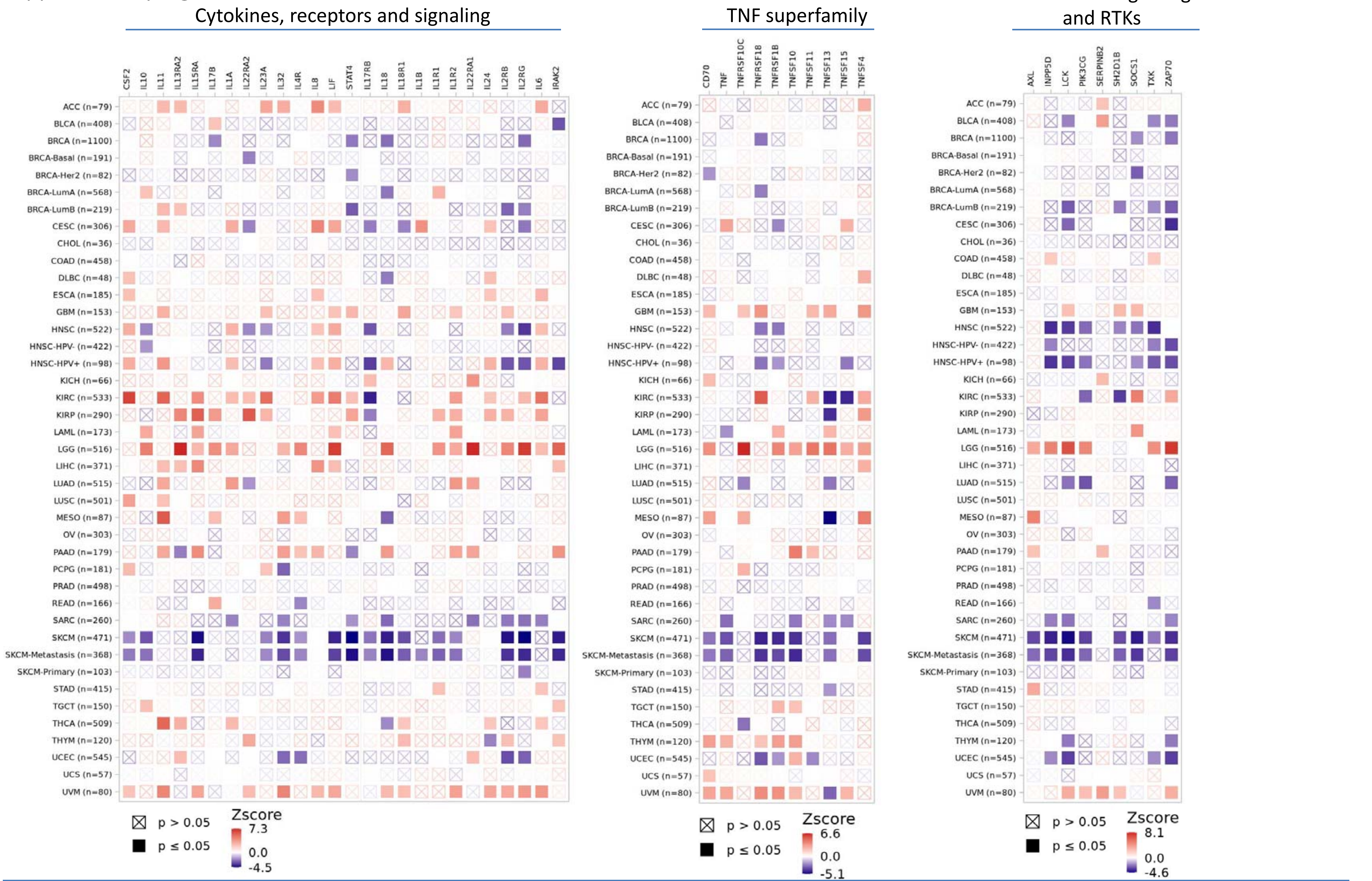

Supplementary Figure S21. Prognostic significance of selected genes in the guadecitabine signatures in 41 TCGA tumor types. Negative Z-score values (coded in blue) indicate decreased risk. Positive Z-score values (coded in red) indicate increased risk.
